# Saikosaponin‑D triggers cancer cell death by targeting the PIM1/c-Myc axis to reprogram oncogenic alternative splicing

**DOI:** 10.1038/s41420-025-02729-w

**Published:** 2025-10-06

**Authors:** Xin Zhang, Xuehui Li, Feng Zhang, Dejun Yang, Qiang Sun, Yuang Wei, Ronglin Yan, Dongliang Xu, Shan Lin, Fuwen Yuan, Weijun Wang

**Affiliations:** 1 Department of Gastrointestinal Surgery, Second Affiliated Hospital of Naval Medical University, Shanghai, China; 2 The Center for Cancer Research, School of Integrative Medicine, Shanghai University of Traditional Chinese Medicine, Shanghai, China; 3 Shanghai TCM-Integrated Hospital, Shanghai University of Traditional Chinese Medicine, Shanghai, China; 4 Department of Pharmacy, Second Affiliated Hospital of Naval Medical University, Shanghai, China; 5 Department of Urology, Shuguang Hospital, Shanghai University of Traditional Chinese Medicine, Shanghai, China

**Keywords:** Drug development, Cancer

## Abstract

Saikosaponins (SSs, including SSA, SSB, SSC, and SSD), the major bioactive compounds in the traditional medicine *Radix Bupleuri*, are emerging agents exhibiting anti-tumor efficacy in several cancers. However, the respective anti-tumor efficacy of these agents and mechanisms in cancers remains unclear. Here, we reported that SSD, among SSs, possessed a significant anti-tumor role across different cancer types in vivo and in vitro by downregulating alternative splicing factors and rewiring oncogenic alternative splicing events. Mechanistically, SSD directly targets PIM1 and blocks the interaction between PIM1 and Myc, and decreases PIM1-mediated Myc phosphorylation at serine 62 and Myc protein stability, resulting in global restraining of Myc-governed alternative splicing factors transcription and inducing oncogenic alternative splicing rewiring. Transcript-specific ablation of SSD-regulated alternative spliced products with CIRSPR-Cas13 or targeting PIM1/Myc with specific small inhibitors significantly desensitizes cancer cells and patient-derived organoids (PDOs) to SSD treatments. These studies demonstrated the potent anti-tumor efficacy of SSD and exposed a PIM1/Myc axis by which SSD modulates the expression of an oncogenic alternative splicing regulatory network that mediates SSD’s anti-tumor role in cancers.

## Introduction

*Radix Bupleuri* (Chaihu) has been a widely used traditional medicine in Asian countries such as China, Japan, and Korea for over two thousand years for its properties, including anti-inflammation, antitumor, antifebrile, antibacterial, antiviral, and neuroprotective effects [1]. Phytochemical studies have demonstrated that Saikosaponins, especially SSA, SSB2, SSC, and SSD, are the main bioactive compounds [2, 3], highlighting their critical roles in *Radix Bupleuri* in clinical settings. Many studies have reported that Saikosaponins possess potent pharmacological effects such as sedation, antipyretic, antiviral, anti-inflammation, and antitumor properties, consistent with the clinical benefits of *Radix Bupleuri* [4, 5]. Saikosaponins in *Radix Bupleuri* are complex compounds composed of triterpene aglycone and a carbohydrate part containing 1–13 monosaccharides [6]. Accumulating studies have reported the anti-tumor efficacy of the main Saikosaponins in *Radix Bupleuri* [7–9], while the individual anti-tumor effects and direct targets remain unclear. Additionally, the different structural characteristics of various Saikosaponins may result in distinct pharmacological activities. Currently, no studies have been reported comparing the differences in anti-tumor effects and cancer type-specific preferences of different Saikosaponins.

Alternative splicing is a tightly regulated process that pre-mRNA undergoes during its maturation, whereby protein-coding segments are assembled in diverse combinations, ultimately generating proteins with distinct amino acid sequences and resulting in diverse or even opposing functions [10]. Dysregulation of alternative splicing has been reported to be implicated in various disease conditions such as cancer, diabetes, aging, hypertension, and mental disorders [11–14]. Specifically, the transcriptomic landscape of cancer reveals cancer-specific splicing alterations, which are often linked to tumor drivers, making them vulnerable therapeutic targets for the treatment of cancer [15]. The oncogenic transcription factor Myc, whose transcription activity is controlled by its phosphorylation, is dysregulated in over 50% of cancers [16] and has been reported to regulate a pan-cancer network of splicing factors and is involved in alternative mRNA splicing in tumorigenesis and progression, such as prostate cancer [17, 18]. The oncogenic PIM1 kinase has been implicated as a cofactor for Myc in carcinogenesis, exerting its tumorigenicity by regulating Myc phosphorylation at S62, which increases Myc stability, thereby enhancing its transcriptional activity [19].

In this study, we investigated the impact of major Saikosaponins derived from *Radix Bupleuri* on different cancer models, including gastric cancer, prostate cancer, and colorectal cancer, and identified SSD as a potential anti-tumor agent with the most significant anti-tumor efficacy by binding to the oncogenic protein PIM1 and promoting PIM1-mediated Myc dephosphorylation and degradation.

## Results

### SSD suppresses cancer cell and organoid growth activity in different cancer types

**Fig. 1:**
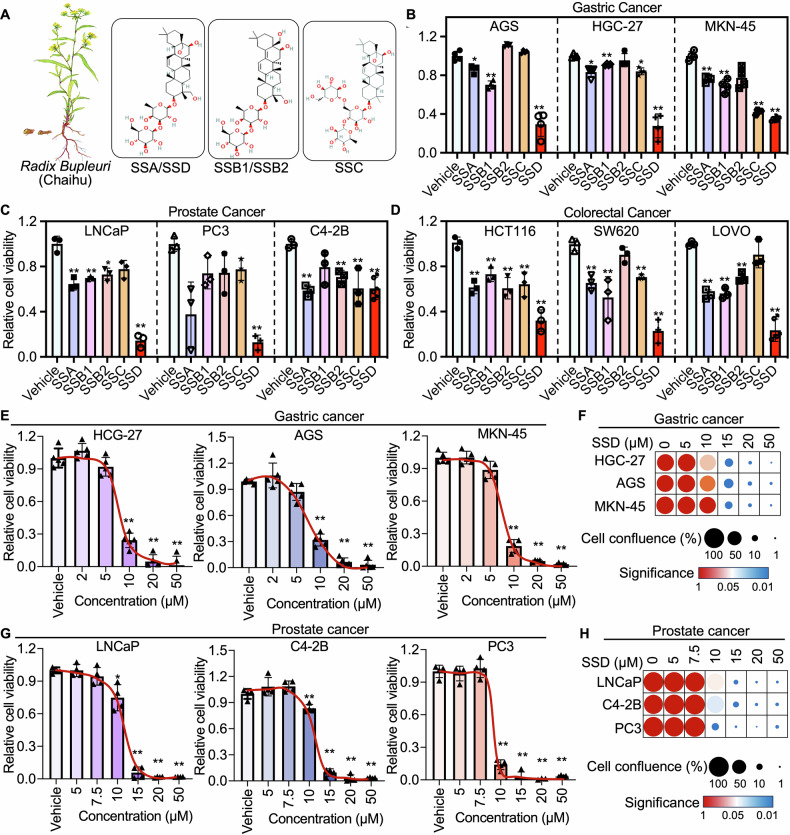
SSD exerted potent anti-tumor growth efficacy in multiple cancer types in vitro. **A** Characters of *Radix Bupleuri* and the structures of SSs, including SSA, SSB1, SSB2, SSC, and SSD. Cell viability was determined with CCK-8 assays after different gastric cancer cells (**B**), prostate cancer cells (**C**), and colorectal cancer cells (**D**) were treated with Vehicle or SSD (10 μM) for 48 h. **E** CCK-8 assays were performed to determine cell viability after gastric cancer cells were treated with different dosages of SSD for 48 h. **F** The cell confluence was recorded with a microscope after gastric cancer cells were treated with different dosages of SSD for 48 h, and the relative cell confluence was calculated with image J. **H**, **I** The cell viability and cell growth of prostate cancer were determined with CCK-8 assays (**G**) and cell confluence analysis, respectively. *, *p* ≤ 0.05, **, *p* ≤ 0.01.

SSA, SSB1, SSB2, SSC, and SSD are the primary active compounds derived from *Radix Bupleuri* (Fig. 1A) that have been reported to play antitumor roles in various cancers. However, the pharmacological effects of different Saikosaponins in cancer remain to be explored. To determine the anti-tumor efficacy of the main Saikosaponins derived from Radix Bupleuri, we assessed the cell growth inhibition efficacy of SSA, SSB1, SSB2, SSC, and SSD in nine cancer cell models, including gastric cancer, prostate cancer, and colorectal cancer cells, respectively. We observed a significant tumor suppression effect of SSD among the different Saikosaponins across different cancer types (Fig. 1B–D). Additional cell viability and growth assays demonstrated that SSD exhibited a dose-dependent tumor growth inhibition effect in both gastric cancer (Fig. 1E, F) and prostate cancer (Fig. 1G, H). These studies collectively demonstrate the superiority of SSD among the main active compounds derived from *Radix Bupleuri* in tumor suppression in various cancers such as gastric cancer, prostate cancer, and colorectal cancer.

**Fig. 2:**
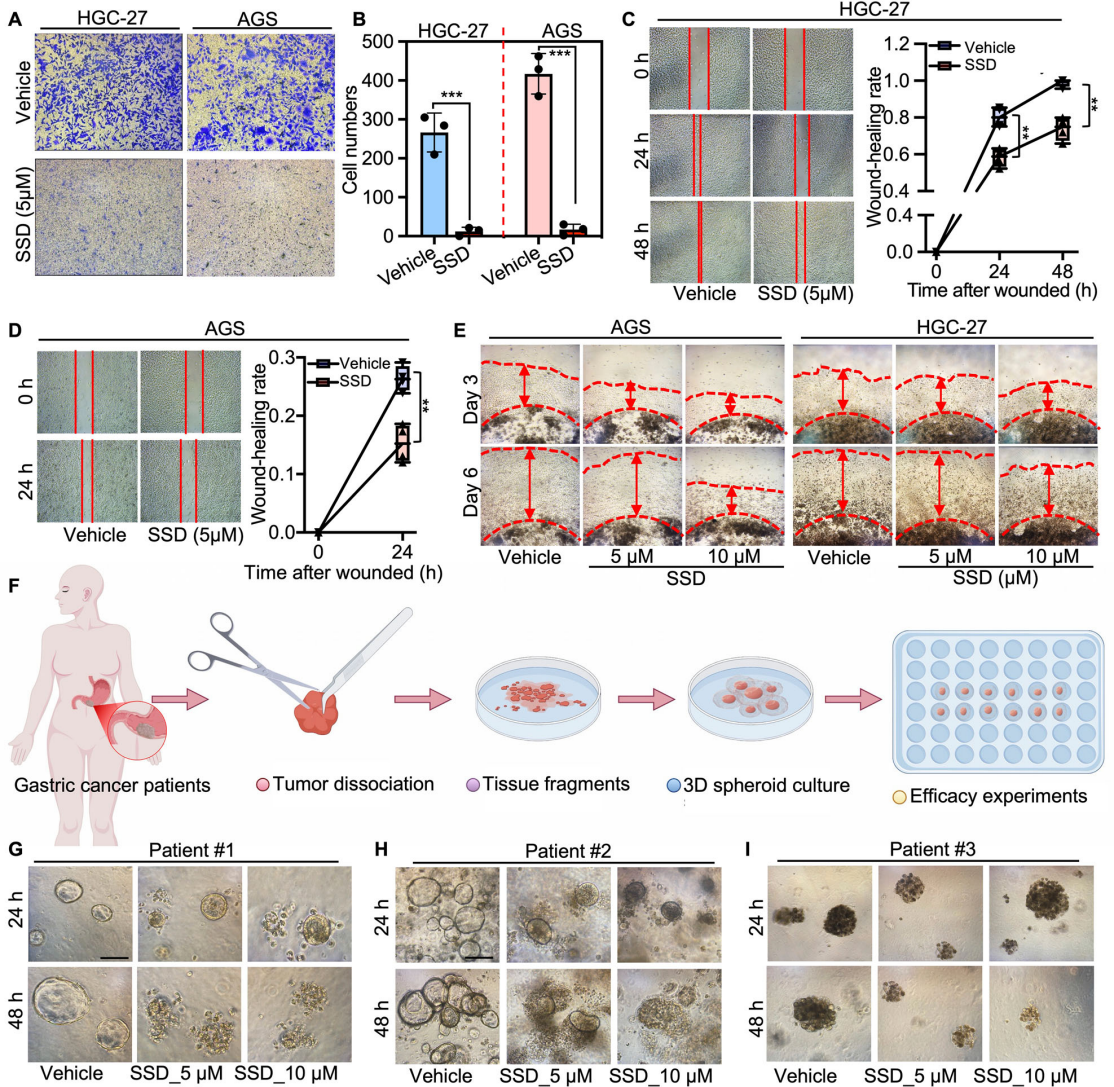
SSD suppressed cancer cell and organoid growth and metastasis in gastric cancer models. **A**, **B** Transwell assays were carried out to evaluate the cell invasion rate after being treated with SSD (5 μM) in HGC-27 and AGS (F) for 24 h. **C**, **D** Wound-healing assays were performed to determine the cell migration after the cells were treated with SSD (5 μM) for the indicated times. **E** 3D Matrigel drop invasion assays after AGS cells and MGC-27 cells were plated in Matrigel drops and treated with SSD at the specified concentrations. Media and treatment were exchanged every 3 days for 6 days. **F** Schematic representation of the experimental design for the generation of PDOs from gastric cancer patients. **G**–**I** The organoid images were taken after being incubated with indicated dosages of SSD for 24 h and 48 h, respectively. ns not significant. **, *p* ≤ 0.01. ***, *p* ≤ 0.001.

We further investigated the role of SSD in cancer cell models by determining the cell survival and migration ability after treatment with different doses of SSD. Our findings revealed that SSD inhibited the colony formation ability of cancer cells in a dose-dependent manner (Fig. S1). Additionally, a low dose of SSD (5 μL), which did not visibly inhibit cell growth, markedly impeded cell invasion and migration as determined by trans-well, wound-healing, and 3D cultured tumor cell spheroid assays (Fig. 2A–E). In conclusion, these data highlighted the significant anti-cancer efficacy of SSD across different types of cancers. Notably, to further evaluate the potential tumor-suppressive role of SSD in clinical settings, we derived three cancer organoids from gastric cancer patients (Fig. 2F). The results indicated that treatment with a low dose of SSD significantly induced cell death in gastric cancer organoids derived from various gastric cancer tissues (Fig. 2G–I).

### SSD decreases alternative splicing factors and rewires alternative splicing

**Fig. 3:**
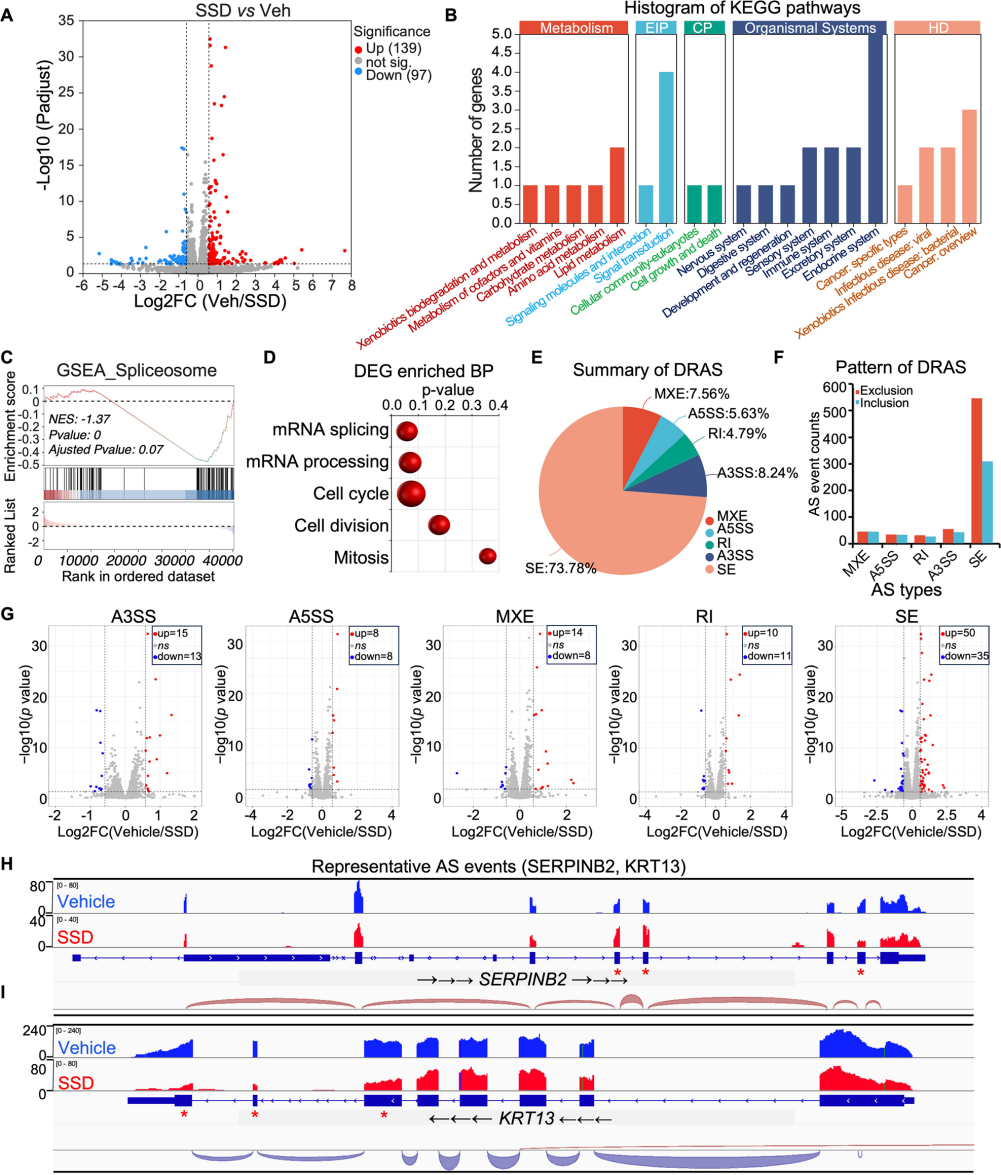
SSD treatment rewires alternative splicing. **A** The volcano plot shows the differentially expressed genes (DEGs) induced by SSD. **B** The gene numbers of DEGs that are regulated by SSD in different biological processes. **C** GSEA analysis of SSD-regulated genes. **D** The pathways enrichment analysis of SSD down-regulated genes. **E**, **F** The summary of different types of SSD differentially regulated splicing events (DRSEs). **G** The volcano plots showed the expression changes of genes regulated by different alternative splicing types, including A3SS, A5SS, MXE, RI, and SE. **H**, **I** Visualization of the representative AS events of *SERPINB2* and *KRT13*.

Encouraged by the in vitro anti-tumor efficacy of SSD in cancer cells and PDOs, we conducted transcriptome analysis to uncover the biological processes regulated by SSD treatment. The result revealed that over 200 genes were differentially regulated by SSD treatment, impacting various cellular processes (Figs. 3A, B and S2A). Interestingly, Gene Set Enrichment Analysis (GSEA) and pathway enrichment analysis of genes downregulated by SSD showed significant enrichment of Spliceosome and mRNA splicing-related pathways such as mRNA splicing and mRNA processing (Fig. 3C, D). This finding prompted us to further investigate the changes and roles of alternative splicing induced by SSD. Following SSD treatment, there were over one thousand alternative splicing events, with skipped exon (SE) accounting for over 70%, involving both exon inclusion and exclusion (Fig. 3E, F).

Specifically, a significant number of the total transcripts of the genes that undergo alternative splicing after SSD treatment were changed, especially the SE type (Fig. 3G). Given the noticeable alternative splicing changes caused by SSD, we suppose these alternative splicing-corresponding genes may contribute to SSD-induced cancer cell growth suppression by modulating the stability of alternative splicing transcripts functionally cortical in cancer progression. To further explore detailed insight into the alternative splicing changes induced by SSD, visualization of RNA-seq within representative genes, including SERPINB2 and KRT13, indeed showed significant exon enrichments and changes of total reads aligned within the chromatin of the indicated genes (Fig. 3H, I). These data together revealed that SSD treatment induced dysregulation of alternative splicing pathways and rewired the alternative splicing landscape in cancer.

### Manipulation of SSD-induced CYP1A1 splicing variants suppresses cancer growth

**Fig. 4:**
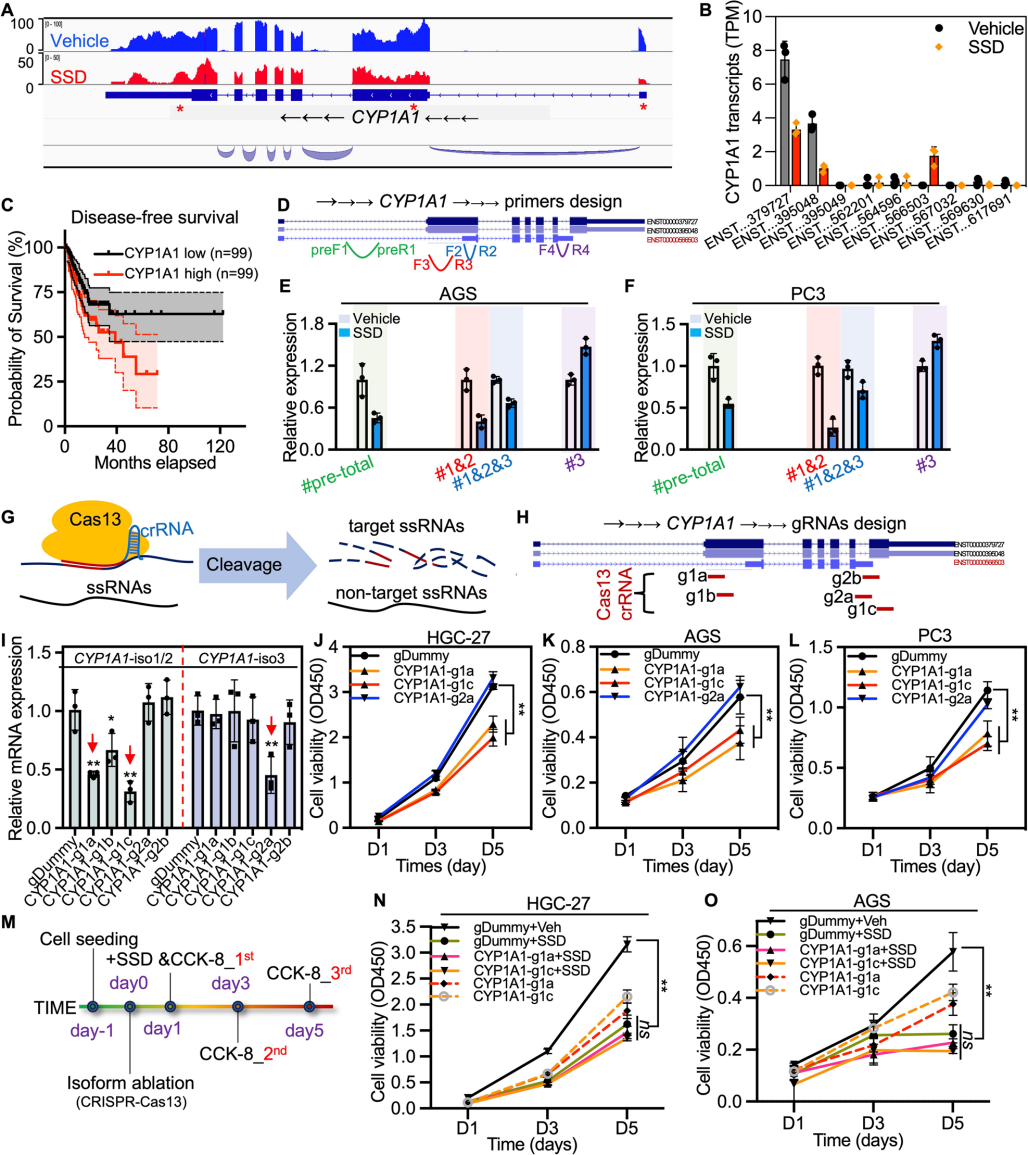
Ablation of SSD-enhanced *CYP1A1* alternative spliced variants impaired SSD-induced cancer cell growth inhibition. **A**, **B** Transcriptome analysis of the TPM of *CYP1A1* different spliced variants after SSD treatment. **C** Disease-free survival (DFS) analysis of the expression of CYP1A1 in the TCGA-STAD cohort. **D** Primer pairs design and qPCR for the detection of premature CYP1A1, as well as three different spliced variants. **E**, **F** RT-qPCR to determine the alternative spliced transcripts of CYP1A1 after SSD treatment in AGS and PC3 cells. **G**, **H** The working model of the CRISPR-Cas13 system and the locations of gRNAs corresponding to CRISPR-Cas13d targeting different *CYP1A1* variants. **I** The deletion efficiency evaluation by qPCR. **J**–**L** The impact of different CYP1A1 spliced variants on HCG-27 cell viability was determined with CCK-8 reagents after indicated *CYP1A1* variants were knocked down with CRISPR-Cas13 for 1 d, 3 d, and 5 d. **M** The diagram indicated the experiment design to determine the impact of *CYP1A1* variant 1 and variant 2 in SSD treatment-induced cell growth inhibition. **N**, **O** CCK-8 assays to detect cell growth after CRISPR-Cas13 transfection or combination with SSD treatment. ns not significant, **, *p* ≤ 0.01.

Given our RNA-seq data revealed that SSD treatment induced significant alternative splicing of *CYP1A1* transcripts (Fig. 4A, B), the established role of *CYP1A1*, particularly transcripts 1 and 2, in cancer progression [20–22], and our clinical finding linking higher *CYP1A1* expression to worse survival in gastric cancer patients (Fig. 4C), we next investigated the role and mechanism of *CYP1A1* alternative splicing in SSD-induced tumor suppression. Primers were designed to specifically evaluate the relative expression of three transcripts as well as the premature mRNA of *CYP1A1* (Fig. 4D), which demonstrated a significant downregulation of total mRNA and transcripts 1/2 of *CYP1A1* after SSD treatments, with enhanced expression of transcripts 3 in both gastric and prostate cancer cell models (Fig. 4E, F). Given the reported functional distinctiveness of different *CYP1A1* transcripts in cancer, as well as the significant differences of *CYP1A1* alternative splicing induced by SSD, we are interested in exploring whether deleting the *CYP1A1* transcripts that are upregulated by SSD can inhibit cancer growth. Several gRNAs designed to target different transcripts of *CYP1A1* corresponding to CRISPR-Cas13 were tested (Fig. 4G–I). Results showed that deletion of transcript 1/2, which was suppressed by SSD, significantly inhibited cancer cell growth (Fig. 4J–L). However, knocking down transcript 3, which was enhanced by SSD, had minimal impact on cancer cell growth (Fig. 4J–L). Furthermore, the tumor growth suppression effect of SSD in cancer cells with *CYP1A1* transcript 1/2 deletion decreased significantly (Fig. 4M–O). Colony formation assays also demonstrated comparable inhibition of tumor survival by either SSD treatment or knockdown of *CYP1A1* transcript 1/2 (Fig. S3). These findings collectively suggest that SSD suppresses tumor growth, at least partially, by influencing the alternative splicing of *CYP1A1* transcripts. Notably, other genes alternatively spliced by SSD treatment may also contribute to its anti-tumor effect, as they are associated with cancer prognosis (Fig. S4). Further research is needed to investigate these additional SSD-regulated splicing events.

### SSD treatment restrains Myc transcription activity on alternative splicing factors

**Fig. 5:**
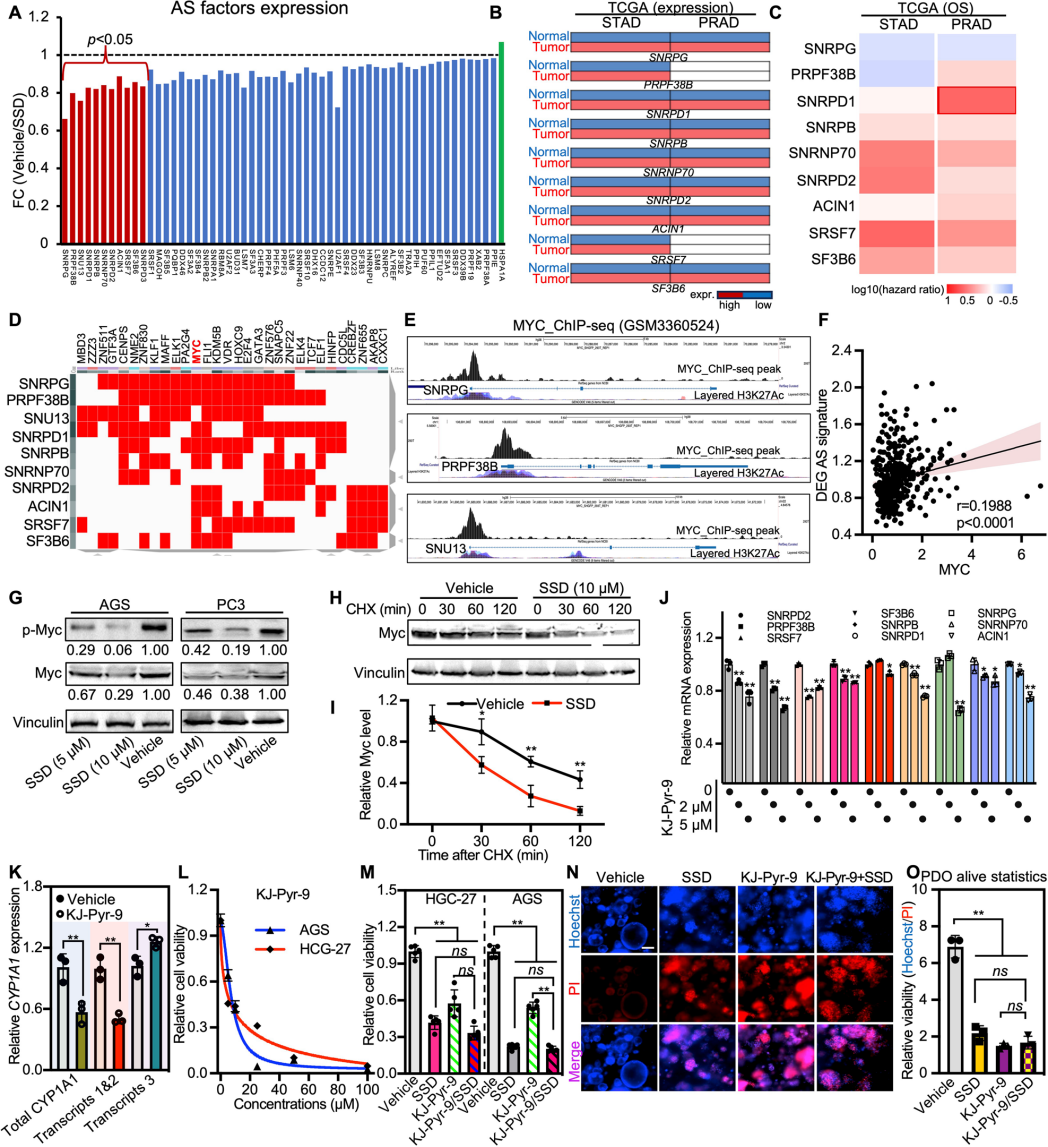
Myc mediated the SSD-induced global alternative splicing factors downregulation. **A** The fold changes (FC) of the alternative splicing factors after being treated with SSD. The relative expression of SSD significantly changed alternative splicing factors in gastric cancer and prostate cancer tissues versus adjacent tissue (**B**) and their correlation with patient survival (**C**) in TCGA cohorts. **D** Target prediction of SSD changed alternative splicing factors with the ChEA3 portal. **E** ChIP-seq visualization of Myc enrichment within promoters of indicated alternative splicing factors. **F** Pearson correlation of Myc and SSD significantly changed the alternative splicing factor signature. **G** Western blot to detect the expression of Myc and p-Myc after cells were treated with indicated dosages of SSD for 24 h. Vinculin was used as a loading control. **H**, **I** Protein half-life assay after cells were treated with Vehicle and SSD to determine the impact of SSD on Myc protein stability. **J** The relative expression of SSD changed alternative splicing factors after being treated with the Myc inhibitor KJ-Pr-9. **K** RT-qPCR to determine the alternative spliced transcripts of CYP1A1 after KJ-Pr-9 treatment in AGS cells. **L** The cell growth inhibition effect of different dosages of KJ-Pr-9 in AGS and HGC-27 cells was determined by CCK-8 assays. The combination effects of SSD and KJ-Pr-9 in cancer cells HGC-27 and AGS (**M**), and the representative gastric cancer patient-derived organoids images (**N**), and their corresponding statistical analysis of the relative cell viability (**O**). ns, not significant. **, *p* ≤ 0.01.

Considering that transcriptome analysis has revealed a significant enrichment of Myc targets (Fig. S2B, C) and splicing (Fig. 3C–F), as well as previous studies have shown that Myc regulates a pan-cancer network of splicing factors and is involved in alternative mRNA splicing in cancers [17, 18], we hypothesized that SSD may regulate the Myc transcription activity and thus modulate the alternative splicing factors expression. Indeed, we observed a global downregulation of alternative splicing factors after SSD treatment, among which, over ten alternative splicing factors were statistically decreased by SSD (Fig. 5A). Interestingly, most of these genes’ expression in gastric cancer and prostate cancer tissues of TCGA cohorts is upregulated, and their enhanced expression indicates worse patients’ prognosis (Fig. 5B, C). Furthermore, transcription factors prediction analysis of regulated alternative splicing factors retrieved Myc as a transcription factor that covered most of the SSD-regulated alternative splicing factors (Fig. 5D). ChIP-seq visualization of Myc further revealed the enrichment of Myc on the promoter of these genes (Figs. 5E and S5). A positive correlation of Myc expression with SSD-regulated alternative splicing factors is also observed in cancer cohorts (Fig. 5F and S6A). Moreover, treatment of SSD had no significant impact on Myc mRNA expression as determined by both RNA-seq and RT-qPCR (Fig. S6B–D), while decreasing both the total expression and phosphorylation of Myc as well as the stability of Myc protein as determined by protein half-life assays (Fig. 5G–I), and inhibition of Myc transcription activity with inhibitor KJ-Pyr-9 further demonstrated a significant downregulated expression of alternative splicing factors (Fig. 5J) and altered CYP1A1 transcripts expression (Fig. 5K). Consistently, cancer cells, as well as gastric cancer organoids treated with Myc inhibitor, lost the sensitivity to SSD compared with wild-type cells (Fig. 5L–O). These results together demonstrate that SSD suppressed Myc transcription activity by down-regulating Myc protein expression and phosphorylation, and is responsible for SSD treatment-mediated alternative splicing reprogramming and tumor growth inhibition.

### SSD binds to PIM1 and decreases PIM1-mediated Myc phosphorylation

**Fig. 6:**
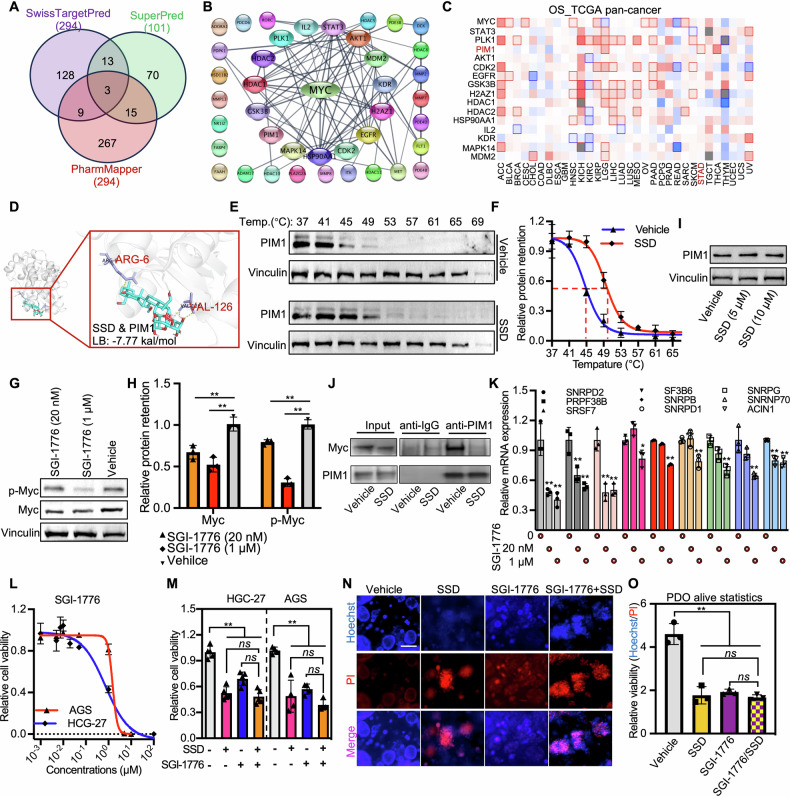
SSD binds to and downregulates PIM1 to suppress Myc transcription activity. **A** The targets of SSD were predicted with SuperRed, SwissTargetPred, and PharmMapper. **B** Protein-protein interaction (PPI) of the top SSD predicted targets and Myc. **C** Overall survival (OS) analysis of top SSD-predicted targets IN TGCA pan-cancer. **D** Molecular docking of PIM1 with SSD. Representative western blot images (**E**) and the statistical analysis (**F**) of PIM1 of CETSA assays after cells were treated with vehicle or SSD. **G**, **H** Western blot analysis of the expression of Myc, p-Myc, and Vinculin (internal control) after cells were treated with indicated dosages of PIM1 inhibitor SGI-1776. **I** Western blot analysis of the expression of PIM1 and Vinculin (internal control) after cells were treated with indicated dosages of SSD. **J** Protein immunoprecipitation assay with PIM1 demonstrated the interaction of PIM1 and Myc after being treated with Vehicle and SSD. **K** The relative expression of SSD changed alternative splicing factors after being treated with the Myc inhibitor SGI-1776. **L** The cell growth inhibition effect of different dosages of SGI-1776 in AGS and HGC-27 cells was determined by CCK-8 assays. The combination effects of SSD and SGI-1776 in cancer cells HGC-27 and AGS (**M**) and gastric cancer patient-derived organoids (**N**, **O**). ns, not significant, *, *p* ≤ 0.05, **, *p* ≤ 0.01.

To further determine the direct binding targets of SSD in suppressing gastric cancer growth, we performed target prediction using three model databases, including SwissTargetPred, SuperPred, and PharmMapper. Targets identified in at least two models were considered to be SSD targets (Fig. 6A and Table S3). Unfortunately, Myc did not appear on the list. However, PPI analysis of SSD targets revealed a close association of 15 targets with Myc (Fig. 6B). Among these, PIM1 exhibited significant oncogenic potential in gastric cancer (Fig. 6C). Molecular docking identified Arg-6 and Val-126 as key mediators of high-affinity SSD binding to PIM1 (Fig. 6D). Mutations at these sites, including the catalytic domain residue Val-126 [23], significantly weakened the interaction (Fig. S7A). Crucially, SSD bound PIM1 has with higher affinity than SSA, SSB, or SSC, demonstrating its specificity (Fig. S7B–E). The following cellular thermal shift assay (CETSA), a method used to identify drug targets in vivo [24], also confirmed the binding of SSD to PIM1 in cancer cells (Fig. 6E, F).

Previous studies have indicated that PIM1 can bind to Myc and regulate its phosphorylation and protein expression [19, 25]. Subsequently, we assessed the total protein and phosphorylated Myc levels after treatment with a PIM1 inhibitor, SGI-1776, which resulted in a significant decrease in Myc levels in both total and phosphorylated forms (Fig. 6G, H). We next ask how SSD-targeted PIM1 regulates Myc phosphorylation and stability. Results showed that SSD treatment did not significantly change the expression of PIM1 at both mRNA and protein levels (Fig. 6I and S8). The protein immunoprecipitation assays revealed that SSD treatment disrupts the PIM1-Myc interaction (Fig. 6J). These data collectively demonstrated that SSD binds PIM1, which inhibits the interaction of PIM1 with Myc and thus reduces Myc phosphorylation and protein stability. Additionally, the supplementation of a PIM1 inhibitor SGI-1776 significantly suppressed the expression of SSD-regulated alternative splicing factors (Fig. 6K) and eliminated the drug sensitivity of cancer cells to SSD (Fig. 6L–O). It should be noted that SGI-1776 could also act as an inhibitor of PIM2 and PIM3, and previous studies indicated that PIM2 can also regulate Myc phosphorylation [19]. Here, we found that only PIM1 was retrieved as the predicted target of SSD among the PIM family (Fig. 6A), and molecular docking indicated the highest binding affinity of SSD with PIM1 compared to PIM2 and PIM3 (Fig. S9A–C). Additionally, the expression of PIM2 was singingly lower in different gastric cancer cell models, either determined by RNA-seq or RT-qPCR (Fig. S9D, E). In conclusion, our findings demonstrate that SSD can directly bind to PIM1, inhibiting Myc transcriptional activity by reducing its phosphorylation and protein stability, leading to the downregulation of alternative splicing factors and the alteration of oncogenic alternative splicing events.

### SSD treatment suppresses cancer growth in the xenografted mouse model

**Fig. 7:**
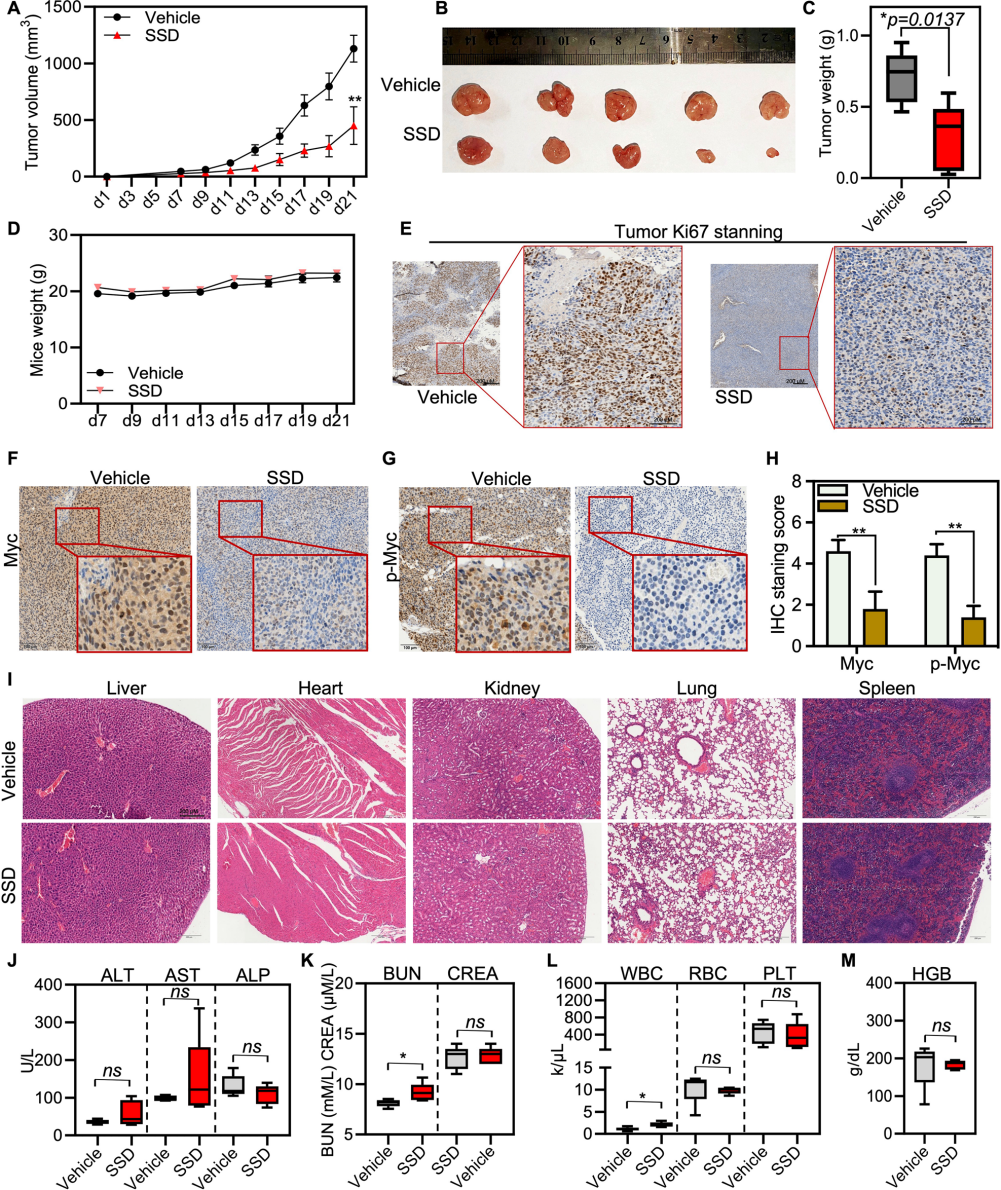
SSD suppressed tumor growth in vivo. **A** The tumor volume was measured every 2 days in mice gavaged with vehicle or SSD (10 mg/kg) (*n* = 5). **B** Tumor images after the cells were executed. **C** The statistical analysis of the tumor mass after the cells were executed. **D** The mice’s weight was measured every 2 days. **E** IHC staining of proliferation marker Ki67 on tumors from mouse models treated with vehicle and SSD, respectively. **F-H** IHC staining of Myc and p-Myc on tumors from mouse models treated with vehicle and SSD, respectively. **I** H&E staining of major organs, including heart, liver, spleen, lung, and kidney. **J**–**M** Statistical analysis of the liver and kidney indicators, as well as blood cells, including ALT, AST, ALP, BUN, CREA, WBC, RBC, PLT, and HGB, after the mice were executed. *n* = 5, ns, not significant, *, *p* ≤ 0.05.

Given the significant anti-tumor efficacy of SSD on cancer cell growth in multiple cell models and cancer organoids, as well as its impact on the oncogenic PIM1-Myc axis in cancer, we further evaluated its anti-tumor efficacy and potential toxicity in vivo. HGC-27 cells were subcutaneously injected into nude mice, and SSD or vehicle was intraperitoneally administered each day. SSD significantly suppressed tumor growth after 2 weeks (Fig. 7A–C), without a significant difference in mouse body weight (Fig. 7D). Immunohistochemistry (IHC) staining demonstrated that SSD treatment reduced Ki67 staining, a proliferation marker, in mouse tumors versus the vehicle group (Fig. 7E). It also significantly lowered both Myc and p-Myc levels (Fig. 7F–H), aligning with in vitro cancer cell results (Fig. 5G). It is noteworthy that hematoxylin and eosin (H&E) staining of major organs, including the liver, kidney, lung, spleen, and heart, revealed no apparent pathological changes after SSD treatment (Fig. 7I). Furthermore, most hematological indexes, such as WBC, RBC, PLT, and HGB, and biochemical indexes of liver and kidney function, including ALT, AST, ALY, BUN, and CREA, were similar between vehicle-treated and SSD-treated mice, except for a slight increase in BUN and WBC levels in the SSD group (Fig. 7J–M). These findings collectively support the anti-tumor efficacy of SSD in vivo without evident toxicity. However, further studies involving different doses of SSD and longer treatment durations are necessary to fully establish the safety profile of SSD in vivo.

## Discussion

*Radix Bupleuri* (also known as Chaihu in China) is a commonly used traditional medicine in Asian countries. Saikosaponins are the primary ingredients and are widely recognized as the bioactive compounds of Radix Bupleuri. Most of the main compounds of Saikosaponins derived from Radix Bupleuri have been reported to exhibit anti-tumor effects in various cancer cell models. However, the specific preference of these homologs in cancer is unclear and requires further assessment. In this study, we systematically evaluated the anti-tumor efficacy of the major compounds of Saikosaponins from Radix Bupleuri in cell models of gastric cancer, prostate cancer, and colon cancer. Our findings demonstrated that SSD had the strongest tumor cell growth inhibition effect among the Saikosaponins tested. Additionally, SSD showed significant anti-tumor effects in gastric cancer organoids and xenografted mice models without causing significant toxicity at the dose that suppressed tumor growth in mice.

It is worth noting that other Saikosaponins, including SSA, SSB1, and SSC, also exhibited statistically significant cancer growth inhibition effects in several cancer cell models (Fig. 1C–E), although not as significant as the effects observed with the same dose of SSD. Further investigations could explore whether these Saikosaponins share the same targets and whether a combination of different Saikosaponins at lower doses could achieve higher anti-tumor efficiency with reduced potential toxicity. STAT3 was also previously reported to be a target of SSD in myoblasts and myotubes [26], but we didn’t observe a significant desensitized effect of STAT3 inhibitors in SSD-induced tumor growth inhibition in cancer cells (Fig. S10). Notably, our network pharmacology analysis indicated that some other proteins may also be targeted by SSD (Fig. 6A), and our transcriptome analysis revealed a significant number of genes and pathways that were differently regulated by SSD treatment. Future omics studies, such as CETSA-MS and Drug affinity responsive target stability (DARTS)-MS, are required to systemically identify the targets, in addition to PIM1, as well as off-targets of SSD in different disease backgrounds.

Dysregulation of alternative splicing is comprehensively involved in tumorigenesis, progression, lineage plasticity, and chemotherapy resistance [11, 15, 27, 28]. There are several strategies to manipulate the oncogenic alternative splicing events for the treatment of abnormal alternative splicing processes in cancer [29]. Small-molecule splicing modulators and antisense oligonucleotides that modulate cancer-relevant alternative splicing decisions are currently in clinical trials. These strategies are highly specific but require further clinical practices to determine if targeting a single alternative splicing event alone is sufficient to address abnormal cell proliferation in cancer clinics without rapidly induced resistance due to the large-scale changes of alternative splicing events during cancer progression. Additionally, targeting alternative splicing factors found to be mutant or whose expressions were dysregulated in different cancers, such as SF3B1, SRSF2, U1, and U2AF1, also holds promise for the treatment of cancers [30–34]. Myc, a well-established oncogene in pan-cancer [35, 36], was found to be functionally critical in regulating a network of co-expressed oncogenic splicing factors in pan-cancer and is necessary for Myc-driven tumorigenesis [17, 37]. However, direct targeting of Myc is currently clinically unsuccessful. Although approaches such as regulating Myc expression or protein stability show preclinical promise, there are no FDA-approved Myc-targeting therapies [17, 38]. Here, we demonstrated that SSD could bind Myc regulator PIM1, thus modulating Myc expression and transcription activity on alternative splicing factors, offering an alternative approach for targeting Myc-regulated alternative splicing in cancer therapy.

In summary, we have identified a PIM1/Myc-regulated alternative splicing process that mediates SSD-induced tumor suppression. Blocking PIM1 or Myc activity with specific inhibitors, or targeting PIM1/Myc-controlled oncogenic alternative splicing events with CRISPR-Cas13, can significantly and effectively inhibit cancer growth, which offers a novel avenue of natural products derived from traditional medicine in suppressing cancers by targeted regulation of Myc-mediated alternative splicing. While our in vivo data show significant tumor suppression, the sample size (*n* = 5) may limit statistical power. Future studies will include larger cohorts, and further experiments are required to determine the potential in vivo toxicity of SSD with long-term treatment. Notably, our target prediction (Fig. 6A) identified other candidates such as AKT1 and MAPK3 as potential SSD interactors. While these may synergize with PIM1 inhibition, our functional assays indicate the PIM1/Myc axis is the primary mediator of SSD’s anti-tumor effects. Future studies are required to determine whether these potential targets are also regulated by SSD and contribute to SSD’s anti-tumor effects. Additionally, while we established that SSD targets the PIM1/Myc axis to exert anti-tumor effects in gastric and prostate cancers, future studies exploring its effects and mechanisms in other cancer types will be valuable for expanding its pan-cancer therapeutic potential.

## Materials and methods

### Cell culture and reagents

Gastric cancer AGS cells were purchased from Yizefeng Biotechnology Co., Ltd. (Shanghai, China) and cultured in Ham’s F12-K (Kaighn’s) with 10% fetal bovine serum (Gibco). HGC-27 and MKN-45 were gifted from Prof. Peike Peng at Shanghai University of Traditional Chinese Medicine and cultured in RPMI 1640 with 10% fetal bovine serum (Gibco). Prostate cancer cells, including LNCaP, C4-2B, and PC3, and colorectal cancer cells, including HCT116, SW620, and LOVO, were maintained in our lab as previously described (20) and were cultured in RPMI 1640 with 10% fetal bovine serum (Gibco). All cell lines were authenticated and tested for mycoplasma contamination. Cells were incubated at 37 °C with 5% CO_2_.

### CCK-8 assays

Cell viability was assessed by the CCK-8 counting kit (GlpBio, America) as we described before [39]. Briefly, cells were planted onto 96-well plates and treated with the specific agents for 72 h CCK-8 reagents were then added and incubated for 1 h. The absorbance (450 nm) was measured with a microplate reader (TECAN Spark, Switzerland).

### Wound-healing assays

The cell migration was determined using wound-healing assays. Cells were plated in six-well plates (1 × 106 cells/well). After 24 h, a linear scratch was generated using a 10 μL pipette tip and incubated within RPMI1640 (1% FBS) with specified drug concentrations. The cell migration was photographed (x10) (LEICA DMi1, Germany Inc.) after 0, 24, and 48 h of wound creation.

### 3D Matrigel drop migration assays

3D Matrigel drop migration assays were performed as previously described [40]. Briefly, a total of 5 × 104 AGS and HGC-27 cells were suspended in 10 μL Matrigel and plated into a 24-well plate as a droplet. After 30 min, 300 μL of medium supplemented with indicated concentrations of SSD was added, and cultured for 6 days. The images were taken on days 3 and 6. DMSO was used as vehicle controls.

### Colony formation assays

The colony assay was performed as we previously described [39]. Briefly, cells (1000 cells/well) were seeded into 6-well plates and cultured for 7 days, supplied with the indicated agents. The cells were fixed with a 4% polyformaldehyde solution (Servicebio, China) and stained with a 1% crystal violet solution (Adamas, China).

### Animal studies

Four-week-old BALB/c male nude mice (mouse weight 20 ± 4 g) were purchased from Shanghai Jihui Laboratory Animal Care Co., Ltd (Shanghai, China) and were housed in a 12 h light/dark cycle, at 22–25 °C and 60% relative humidity with free access to water and food. After the mice were allowed to acclimate for 1 week, 2 × 10^6^ HGC-27 cells in 100 μL sterile PBS were subcutaneously injected. The mice were randomly divided into two groups and were intraperitoneally injected with 200 μL vehicle or SSD (10 mg/kg) each day, respectively. The mice’s weight and tumor volume were measured every 2 days. All the animal experimental procedures followed the National Guidelines for Animal Usage in Research (China) and were approved by the Ethics Committee of Shanghai University of Traditional Chinese Medicine (Ethics NO. PZSHUTCM2309080005).

### H&E staining and immunohistochemistry (IHC) staining of Ki67

Mice organs, including the heart, lung, liver, and kidney, were fixed with 4% Paraformaldehyde for 48 h and embedded with paraffin. Paraffin sections of organs were stained with Hematoxylin and Eosin Staining Kit (H&E kit) (60524ES60, Yeasen Biotechnology (Shanghai) Co., Ltd, China) after dehydration with ethanol and xylene. For IHC staining of Ki67, Dewaxed tissue slides in xylene and rehydrated with graded ethanol to water and oaked in 3% H_2_O_2_ for 20 min. Antigen retrieval was conducted in citrate buffer (pH 6) at 95 °C for 20 min, and the tissues were incubated with 5% BSA at room temperature for 45 min. The slides were incubated in Ki67 antibody (Servicebio GB121141, diluted 1:500) in a wet box overnight at 4 °C, and then incubated with the secondary antibody for 60 min at room temperature. DAB Horseradish Peroxidase Color Development Kit (Beyotime P0203) was then applied following the manufacturer’s instructions. Counterstain nuclei with hematoxylin (H&E kit, Yeasen 60524ES60). The slides were examined with a PreciPoint M8 Microscope (PreciPoint M8 Microscope and Scanner, Freising, Germany).

### Target prediction and molecular docking

The targets of SSD were obtained from SwissTargetPrediction (http://www.swissdock.ch/). Molecular docking of SSD with PIM1 was performed as described previously [41]. Briefly, the SSD’s SDF structure was retrieved from PubChem (https://pubchem.ncbi.nlm.nih.gov/) and converted to mol2 format using Chem 3D software. The PIM1 structure was downloaded from RCSB (https://www.rcsb.org/). PyMol was used for pre-docking SSD and PIM1. The processed structure was then saved in PDBQT format. The binding properties of SSD to PIM1 were analyzed with Autodock Vina.

### RNA-seq and transcriptome analysis

Total RNA was extracted with FastPure RNA Isolation Kit (Vazyme RC112-01, China) from AGS cells after being treated with 5 μM SSD for 24 h. The RNA quality was checked by Bioanalyzer 2100 (Aligent, USA), and samples with an integrity number (RIN) above 9 were selected for RNA-seq library generation as we described before [42]. RNA-seq was performed by Majorbio Co., Ltd. (China). Data analysis was conducted on the online tool of Majorbio Cloud Platform (https://cloud.majorbio.com/page/tools/). Raw data were deposited in GEO with the accession number GSE277023.

### Establishment of patient-derived gastric cancer organoids and viability assays

For the generation of gastric cancer organoids, the gastric cancer tissues were obtained from surgically resected specimens and confirmed with H&E staining by the Department of Pathology of the Second Affiliated Hospital of Naval Medical University. The tumor tissues were sent to Shanghai JFKR Organoid Biotechnology Co., Ltd to establish the organoids. All organoids were cultured in the gastric cancer organoid-specific medium (JFKR-GC-100, Shanghai, JFKR). For the organoid viability assay, organoids were maintained in fresh gastric cancer organoid-specific medium containing the indicated agents for 24 h or 48 h. The organoids’ morphology and viability were detected by brightfield imaging and staining with PI and Hoechst fluorescence dye. All procedures performed in studies involving gastric cancer patient samples were in accordance with the ethical standards of the Second Affiliated Hospital of Naval Medical University research committee and with the 1964 Helsinki Declaration and its later amendments or comparable ethical standard, and ethical approval was obtained from the Ethics Committee of the Second Affiliated Hospital of Naval Medical University (Approval No. 2023SL006). Informed consent was obtained from all subjects.

### RNA extraction and quantitative real-time PCR (qRT-PCR)

Total RNA was extracted using the FastPure Isolation Kit (Vazyme RC112-01, China). The first strand cDNA was reverse-transcribed with the HiScript II Q RT SuperMix (Vazyme RC223-01, China) and amplified with the ChamQ Universal SYBR qPCR Master Mix (Vazyme Q711-02, China). The indicated gene expression was analyzed as we described before [41] in the LightCycler 480 II machine (Roche). The primers used are listed in Table S1.

### Western blot and protein immunoprecipitation

Cells treated with the indicated conditions were collected and washed with ice-cold PBS three times. RIPA lysis buffer (Beyotime P0013B, China) containing protease and phosphatase inhibitors (Beyotime P1045, China) was then added and incubated on ice for 30 min, vortexed for 15 s every 10 min. The protein was collected after centrifugation for 30 min at 12,000 rpm at 4 °C and boiled at 100 °C for 10 min with 5x loading buffer. Samples were then subjected to SDS-PAGE analysis as previously described [42]. The primary antibodies, including PIM1 (A14210; dilution 1:1000; ABclonal, Wuhan, China), Myc (A1309; dilution 1:2000; ABclonal, Wuhan, China), Phospho-c-Myc-S62 Rabbit pAb (AP0082; dilution 1:2000; ABclonal, Wuhan, China), Vinculin (F0110; dilution 1:2000; Selleck, Houston, USA), and ACTB (GB15001, 1:2000, Servicebio, Wuhan, China), were used at the indicated concentrations. Full and uncropped western blots have been uploaded as “Supplemental Material”. The protein immunoprecipitation assay was performed as described previously [43] with the indicated antibodies.

### CRISPR-Cas13 mediated CYP1A1 transcripts silencing

Five pairs of guide RNA (gRNA) oligos targeting different alternative splicing transcripts were designed and ordered from General Bio. The gRNAs were then cloned into pXR003 (Addgene, 109053). The gRNA and CRISPR-Cas13 plasmids were transfected into cells for 48 h, and total RNA was extracted for qRT-PCR to determine the ablation efficiency of the corresponding gRNAs. The gRNA oligos are listed in Table S2.

### Protein stability assays

To determine Myc stability after SSD treatment, cells were plated in 6-well plates and treated with vehicle or SSD (10 μM) for 24 h. And 20 μg/mL of cycloheximide (DRE-C11830000, Dr.E) was added and incubated for the indicated time (0, 30, 60, and 90 min). Cells were then collected for protein extraction for immunoblotting with Myc and Vinculin antibodies.

### Cellular thermal shift assay (CETSA)

CETSAs were performed as previously described [44]. Briefly, cells were treated with SSD or DMSO for 6 h and then subjected to heat at 37 °C, 41 °C, 45 °C, 49 °C, 53 °C, 57 °C, 61 °C, and 63 °C for 4 min. Subsequently, the cells were lysed by freezing and defrosting with liquid nitrogen and a 25 °C heater for 3 cycles, followed by centrifugation for 20 min at 12,000 rpm at 4 °C. The samples were then analyzed using western blotting to determine the expression levels of PIM1 and Vinculin.

### Statistical analysis

The results are represented as the mean ± standard deviation (S.D.) of at least three independent experiments, and statistical analyses were conducted with Prism (Graphpad Software Inc.). The two-tailed Student’s *t*-test or a nonparametric 1-way analysis of variance (ANOVA) test was used to demonstrate statistical significance. Statistical significance was considered for *p* < 0.05.

## Supplementary information

Supplemental Figs. 1–10 and Tables S1–3

Raw Image for WB

## Data Availability

The data that support the findings of this study are available in the supplementary material. The RNA-seq raw data in this study are available at GEO under accession number GSE277023.

## References

[1] Yang F, Dong X, Yin X, Wang W, You L, Ni J. Radix Bupleuri: a review of traditional uses, botany, phytochemistry, pharmacology, and toxicology. Biomed Res Int. 2017;2017:7597596. 28593176 10.1155/2017/7597596 PMC5448051

[2] Tian RT, Xie PS, Liu HP. Evaluation of traditional Chinese herbal medicine: Chaihu (Bupleuri Radix) by both high-performance liquid chromatographic and high-performance thin-layer chromatographic fingerprint and chemometric analysis. J Chromatogr A. 2009;1216:2150–5. 19084233 10.1016/j.chroma.2008.10.127

[3] Huang HQ, Zhang X, Xu ZX, Su J, Yan SK, Zhang WD. Fast determination of saikosaponins in Bupleurum by rapid resolution liquid chromatography with evaporative light scattering detection. J Pharm Biomed Anal. 2009;49:1048–55. 19201128 10.1016/j.jpba.2009.01.011

[4] Jiang H, Yang L, Hou A, Zhang J, Wang S, Man W, et al. Botany, traditional uses, phytochemistry, analytical methods, processing, pharmacology and pharmacokinetics of Bupleuri Radix: a systematic review. Biomed Pharmacother. 2020;131:110679. 32858498 10.1016/j.biopha.2020.110679

[5] Ashour ML, Wink M. Genus Bupleurum: a review of its phytochemistry, pharmacology and modes of action. J Pharm Pharmacol. 2011;63:305–21. 21749378 10.1111/j.2042-7158.2010.01170.x PMC7197585

[6] Li XQ, Song YN, Wang SJ, Rahman K, Zhu JY, Zhang H. Saikosaponins: a review of pharmacological effects. J Asian Nat Prod Res. 2018;20:399–411. 29726699 10.1080/10286020.2018.1465937

[7] Xiao LX, Zhou HN, Jiao ZY. Present and future prospects of the anti-cancer activities of saikosaponins. Curr Cancer Drug Targets. 2022;23:2–14. 35946101 10.2174/1568009622666220806121008

[8] Li X, Li X, Huang N, Liu R, Sun R. A comprehensive review and perspectives on pharmacology and toxicology of saikosaponins. Phytomedicine. 2018;50:73–87. 30466994 10.1016/j.phymed.2018.09.174 PMC7126585

[9] Lei C, Gao Z, Lv X, Zhu Y, Li R, Li S. Saikosaponin-b2 inhibits primary liver cancer by regulating the STK4/IRAK1/NF-kappaB Pathway. Biomedicines. 2023;11:2859 37893233 10.3390/biomedicines11102859 PMC10604266

[10] Marasco LE, Kornblihtt AR. The physiology of alternative splicing. Nat Rev Mol Cell Biol. 2023;24:242–54. 36229538 10.1038/s41580-022-00545-z

[11] Sciarrillo R, Wojtuszkiewicz A, Assaraf YG, Jansen G, Kaspers GJL, Giovannetti E, et al. The role of alternative splicing in cancer: From oncogenesis to drug resistance. Drug Resist Updat. 2020;53:100728. 33070093 10.1016/j.drup.2020.100728

[12] Wright CJ, Smith CWJ, Jiggins CD. Alternative splicing as a source of phenotypic diversity. Nat Rev Genet. 2022;23:697–710. 35821097 10.1038/s41576-022-00514-4

[13] Tao Y, Zhang Q, Wang H, Yang X, Mu H. Alternative splicing and related RNA binding proteins in human health and disease. Signal Transduct Target Ther. 2024;9:26. 38302461 10.1038/s41392-024-01734-2 PMC10835012

[14] Kim BM. The role of saikosaponins in therapeutic strategies for age-related diseases. Oxid Med Cell Longev. 2018;2018:8275256. 29849917 10.1155/2018/8275256 PMC5924972

[15] Bradley RK, Anczukow O. RNA splicing dysregulation and the hallmarks of cancer. Nat Rev Cancer. 2023;23:135–55. 36627445 10.1038/s41568-022-00541-7 PMC10132032

[16] Lourenco C, Resetca D, Redel C, Lin P, MacDonald AS, Ciaccio R, et al. MYC protein interactors in gene transcription and cancer. Nat Rev Cancer. 2021;21:579–91. 34188192 10.1038/s41568-021-00367-9

[17] Urbanski L, Brugiolo M, Park S, Angarola BL, Leclair NK, Yurieva M, et al. MYC regulates a pan-cancer network of co-expressed oncogenic splicing factors. Cell Rep. 2022;41:111704. 36417849 10.1016/j.celrep.2022.111704 PMC9731204

[18] Phillips JW, Pan Y, Tsai BL, Xie Z, Demirdjian L, Xiao W, et al. Pathway-guided analysis identifies Myc-dependent alternative pre-mRNA splicing in aggressive prostate cancers. Proc Natl Acad Sci USA. 2020;117:5269–79. 32086391 10.1073/pnas.1915975117 PMC7071906

[19] Zhang Y, Wang Z, Li X, Magnuson NS. Pim kinase-dependent inhibition of c-Myc degradation. Oncogene. 2008;27:4809–19. 18438430 10.1038/onc.2008.123

[20] Bauer M, Herbarth O, Rudzok S, Schmucking E, Muller A, Aust G, et al. Diversity of common alternative splicing variants of human cytochrome P450 1A1 and their association to carcinogenesis. Int J Oncol. 2007;31:211–8. 17549424

[21] Chua MS, Kashiyama E, Bradshaw TD, Stinson SF, Brantley E, Sausville EA, et al. Role of Cyp1A1 in modulation of antitumor properties of the novel agent 2-(4-amino-3-methylphenyl)benzothiazole (DF 203, NSC 674495) in human breast cancer cells. Cancer Res. 2000;60:5196–203. 11016648

[22] Okino ST, Pookot D, Li LC, Zhao H, Urakami S, Shiina H, et al. Epigenetic inactivation of the dioxin-responsive cytochrome P4501A1 gene in human prostate cancer. Cancer Res. 2006;66:7420–8. 16885337 10.1158/0008-5472.CAN-06-0504

[23] Zhao Y, Aziz AUR, Zhang H, Zhang Z, Li N, Liu B. A systematic review on active sites and functions of PIM-1 protein. Hum Cell. 2022;35:427–40. 35000143 10.1007/s13577-021-00656-3

[24] Tu Y, Tan L, Tao H, Li Y, Liu H. CETSA and thermal proteome profiling strategies for target identification and drug discovery of natural products. Phytomedicine. 2023;116:154862. 37216761 10.1016/j.phymed.2023.154862

[25] Zhao B, Liu L, Mao J, Zhang Z, Wang Q, Li Q. PIM1 mediates epithelial-mesenchymal transition by targeting Smads and c-Myc in the nucleus and potentiates clear-cell renal-cell carcinoma oncogenesis. Cell Death Dis. 2018;9:307. 29472550 10.1038/s41419-018-0348-9 PMC5833424

[26] Chen LL, Xia LY, Zhang JP, Wang Y, Chen JY, Guo C, et al. Saikosaponin D alleviates cancer cachexia by directly inhibiting STAT3. Phytother Res. 2023;37:809–19. 36447385 10.1002/ptr.7676

[27] Boumpas P, Merabet S, Carnesecchi J. Integrating transcription and splicing into cell fate: transcription factors on the block. Wiley Interdiscip Rev RNA. 2023;14:e1752. 35899407 10.1002/wrna.1752

[28] Venkataramany AS, Schieffer KM, Lee K, Cottrell CE, Wang PY, Mardis ER, et al. Alternative RNA splicing defects in pediatric cancers: new insights in tumorigenesis and potential therapeutic vulnerabilities. Ann Oncol. 2022;33:578–92. 35339647 10.1016/j.annonc.2022.03.011 PMC12361925

[29] Bonnal SC, Lopez-Oreja I, Valcarcel J. Roles and mechanisms of alternative splicing in cancer—implications for care. Nat Rev Clin Oncol. 2020;17:457–74. 32303702 10.1038/s41571-020-0350-x

[30] Graubert TA, Shen D, Ding L, Okeyo-Owuor T, Lunn CL, Shao J, et al. Recurrent mutations in the U2AF1 splicing factor in myelodysplastic syndromes. Nat Genet. 2011;44:53–57. 22158538 10.1038/ng.1031 PMC3247063

[31] Gangat N, Mudireddy M, Lasho TL, Finke CM, Nicolosi M, Szuber N, et al. Mutations and prognosis in myelodysplastic syndromes: karyotype-adjusted analysis of targeted sequencing in 300 consecutive cases and development of a genetic risk model. Am J Hematol. 2018;93:691–7. 29417633 10.1002/ajh.25064

[32] Jafari PA, Sadeghian MH, Miri HH, Sadeghi R, Bagheri R, Lavasani S, et al. Prognostic significance of SF3B1 mutations in patients with myelodysplastic syndromes: a meta-analysis. Crit Rev Oncol Hematol. 2020;145:102832. 31812130 10.1016/j.critrevonc.2019.102832

[33] Hou HA, Liu CY, Kuo YY, Chou WC, Tsai CH, Lin CC, et al. Splicing factor mutations predict poor prognosis in patients with de novo acute myeloid leukemia. Oncotarget. 2016;7:9084–101. 26812887 10.18632/oncotarget.7000 PMC4891028

[34] Wu L, Song L, Xu L, Chang C, Xu F, Wu D, et al. Genetic landscape of recurrent ASXL1, U2AF1, SF3B1, SRSF2, and EZH2 mutations in 304 Chinese patients with myelodysplastic syndromes. Tumour Biol. 2016;37:4633–40. 26508027 10.1007/s13277-015-4305-2

[35] Sanchez-Vega F, Mina M, Armenia J, Chatila WK, Luna A, La KC, et al. Oncogenic signaling pathways in the Cancer Genome Atlas. Cell. 2018;173:321–37. e310. 29625050 10.1016/j.cell.2018.03.035 PMC6070353

[36] Schaub FX, Dhankani V, Berger AC, Trivedi M, Richardson AB, Shaw R, et al. Pan-cancer alterations of the MYC oncogene and its proximal network across the Cancer Genome Atlas. Cell Syst. 2018;6:282–300. e282. 29596783 10.1016/j.cels.2018.03.003 PMC5892207

[37] Hsu TY, Simon LM, Neill NJ, Marcotte R, Sayad A, Bland CS, et al. The spliceosome is a therapeutic vulnerability in MYC-driven cancer. Nature. 2015;525:384–8. 26331541 10.1038/nature14985 PMC4831063

[38] Chen H, Liu H, Qing G. Targeting oncogenic Myc as a strategy for cancer treatment. Signal Transduct Target Ther. 2018;3:5. 29527331 10.1038/s41392-018-0008-7 PMC5837124

[39] Chen Y, Zhou Q, Hankey W, Fang X, Yuan F. Second generation androgen receptor antagonists and challenges in prostate cancer treatment. Cell Death Dis. 2022;13:632. 35864113 10.1038/s41419-022-05084-1 PMC9304354

[40] Li P, Lin Q, Sun S, Yang N, Xia Y, Cao S, et al. Inhibition of cannabinoid receptor type 1 sensitizes triple-negative breast cancer cells to ferroptosis via regulating fatty acid metabolism. Cell Death Dis. 2022;13:808. 36130940 10.1038/s41419-022-05242-5 PMC9492666

[41] Chen Y, Zhou Q, Zhang H, Xu L, Lu L, Shu B, et al. Qingdai decoction suppresses prostate cancer growth in lethal-stage prostate cancer models. J Ethnopharmacol. 2023;308:116333. 36863640 10.1016/j.jep.2023.116333

[42] Yuan F, Hankey W, Wu D, Wang H, Somarelli J, Armstrong AJ, et al. Molecular determinants for enzalutamide-induced transcription in prostate cancer. Nucleic Acids Res. 2019;47:10104–14. 31501863 10.1093/nar/gkz790 PMC6821169

[43] Yuan F, Zhang Y, Ma L, Cheng Q, Li G, Tong T. Enhanced NOLC1 promotes cell senescence and represses hepatocellular carcinoma cell proliferation by disturbing the organization of nucleolus. Aging Cell. 2017;16:726–37. 28493459 10.1111/acel.12602 PMC5506443

[44] Alshareef A, Zhang HF, Huang YH, Wu C, Zhang JD, Wang P, et al. The use of cellular thermal shift assay (CETSA) to study Crizotinib resistance in ALK-expressing human cancers. Sci Rep. 2016;6:33710. 27641368 10.1038/srep33710 PMC5027386

