# Supplementary material for: Saikosaponin‑D triggers cancer cell death by targeting the PIM1/c-Myc axis to reprogram oncogenic alternative splicing
Source: Cell Death Discov. 2025 Oct 6;11:427. doi: 10.1038/s41420-025-02729-w (PMC12501015; doi:10.1038/s41420-025-02729-w)
Supplement: Supplementary file 1 — Supplemental Figs. 1–10 and Tables S1–3 [file 41420_2025_2729_MOESM1_ESM.docx]

**Supplemental materials**

**Saikosaponin‑D triggers cancer death by targeting the PIM1/c-Myc axis to** **rewire oncogenic alternative splicing**

Xin Zhang^1,6^, Xuehui Li^2,3,6^, Feng Zhang^4,6^, Dejun Yang^1^, Qiang Sun^1^, Yuang Wei^2,5^, Ronglin Yan^1^, Dongliang Xu^5^, Shan Lin^3^, Fuwen Yuan^2,🖂^, Weijun Wang^1,🖂^

^1^Department of Gastrointestinal Surgery, Second Affiliated Hospital of Naval Medical University, Shanghai 200003, China

^2^The Center for Cancer Research, School of Integrative Medicine, Shanghai University of Traditional Chinese Medicine, Shanghai 201203, China.

^3^Shanghai TCM-Integrated Hospital, Shanghai University of Traditional Chinese Medicine, Shanghai 200082, China.

^4^Department of Pharmacy, Second Affiliated Hospital of Naval Medical University, Shanghai 200003, China

^5^Department of Urology, Shuguang Hospital, Shanghai University of Traditional Chinese Medicine, Shanghai 201203, China.

**^#^** Correspondence authors. Fuwen Yuan. Tel: +86 18310610831; Email: yuanfuwen@shutcm.edu.cn. Weijun Wang, Email: 610207494@qq.com.

**This PDF file includes:**

Figures S1 to 10

Tables S1 to S3

**Supplemental Figures**

**Figure S1**

**Figure S1. SSD suppressed the colony formation ability of cancer cells.** Cells seeded in 6-well plates were treated with indicated doses of SSD and cultured in for 7 days, colonies were stained and counted. n=3, ns, not significant, **, p ≤ 0.01.

**Figure S2**

**Figure S2. Gene ontology and GSEA analysis of SSD-regulated genes.** (A) Gene ontology analysis was employed to character the biological impacts of SSD treatment in cancer cells. BP, biological process; CC, cellular compound; MF, molecular function. (B and C) GSEA analysis was performed to determine the SSD treatment differentially regulated genes enriched pathways. The terms “Myc target version 2” and “Myc target version 1” rank top among all hallmarks gene sets.

**Figure S3**

**Figure S3. Ablation of CYP1A1 transcript 1/2 suppressed the colony formation ability of cancer cells.** Cells were transfected with CRISPR-Cas13 targeting CYP1A1 transcript 1/2 and transcript 3 respectively. And cultured in 6 well plates for 7 days, colonies were stained and counted. n=3, **, p ≤ 0.01.

**Figure S4**


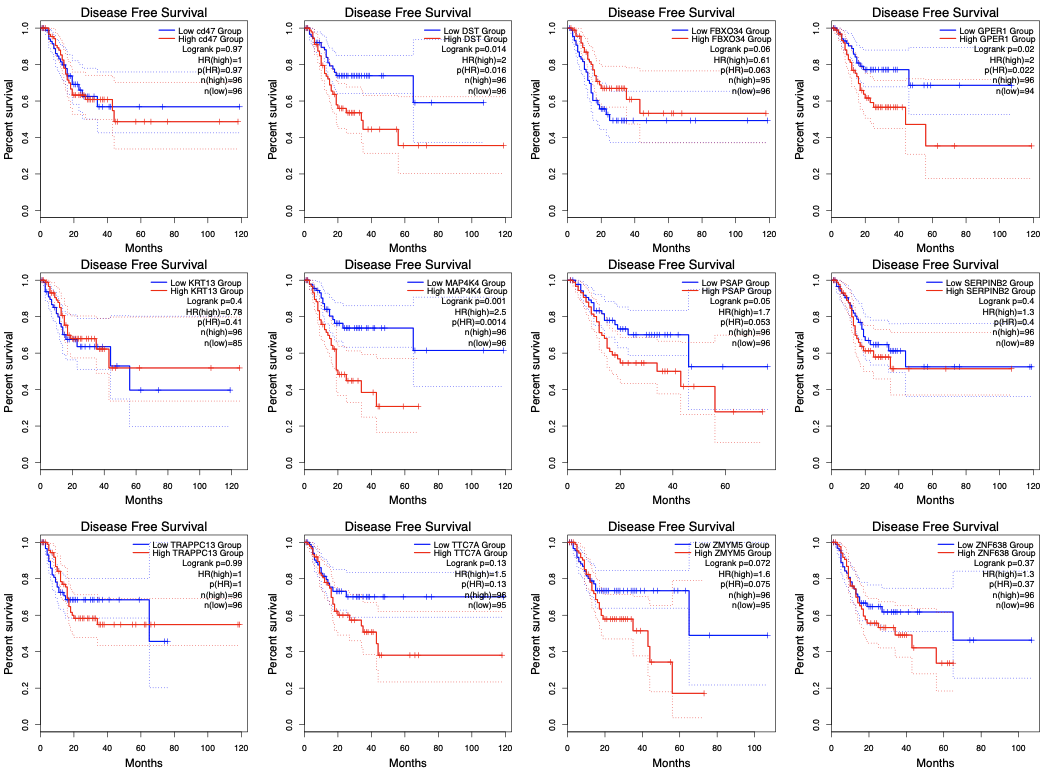


**Figure S4.** **Survival analysis of SSD treatment-regulated AS genes in TCGA gastric cancer patients**. The survival analysis was conducted in the Gepia2 platform (http://gepia2.cancer-pku.cn/#index).

**Figure S5**

**Figure S5. Myc is enriched within the promoter of alternative splicing factors.** The enrichment of Myc on the promoters of specific alternative splicing factors was retrieved from GSM3360524 and visualized with the UCSC Genome Browser.

**Figure S6**


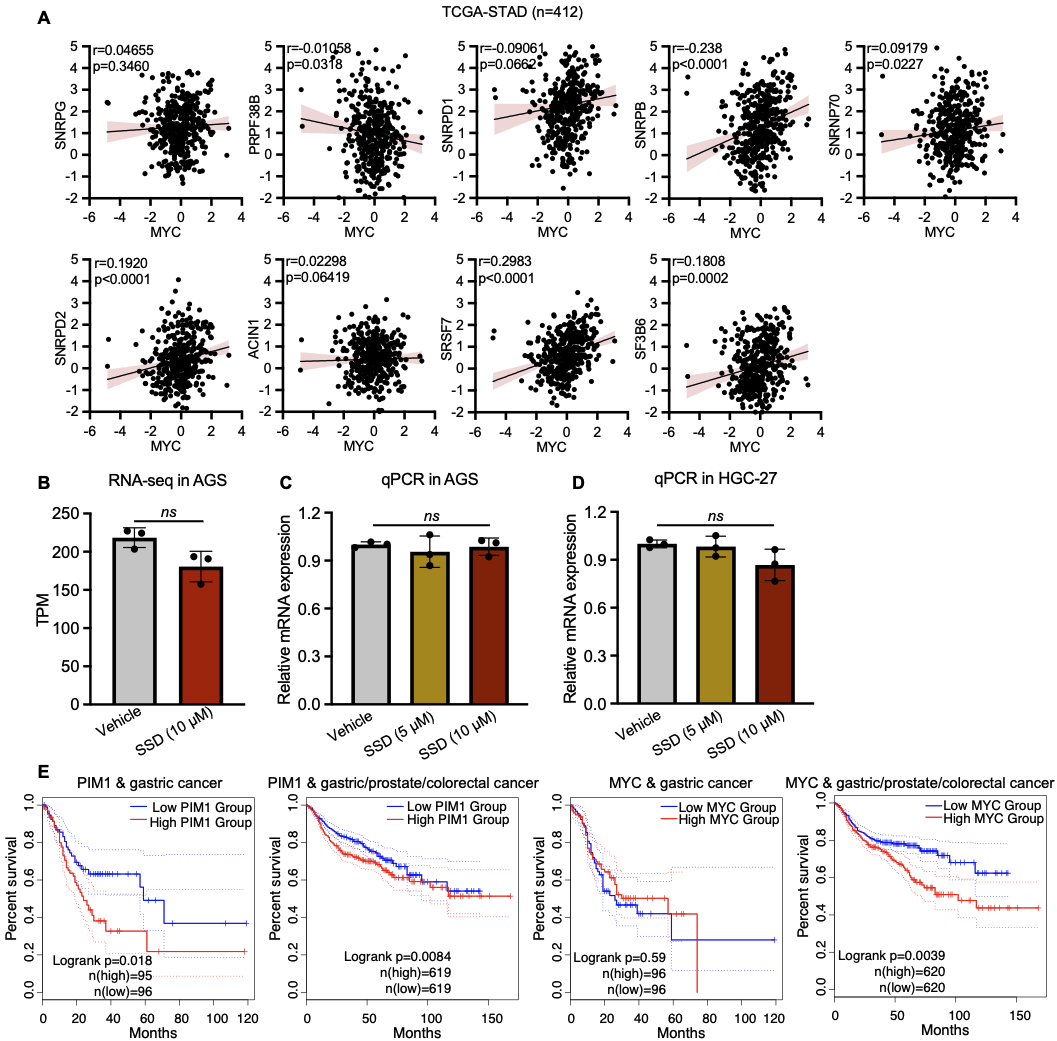


**Figure S6. Expression correlation of Myc and alternative splicing factors in the TCGA-STAD cohort.** (A) The processed mRNA expression data of MYC and specific alternative splicing factors in the TCGA-STAD cohort cancer tissues were downloaded from cBioPortal, and the expression correlation was visualized with Prism software. (B-D) The reflective expression of Myc in AGS and HGC-27 cancer cells was detected by RNA-seq and qPCR. (E) Survival analysis of Myc and PIM1 in TCGA gastric cancer, prostate cancer, and colorectal cancer patients. The survival analysis was conducted in the Gepia2 platform (http://gepia2.cancer-pku.cn/#index).

**Figure S7**


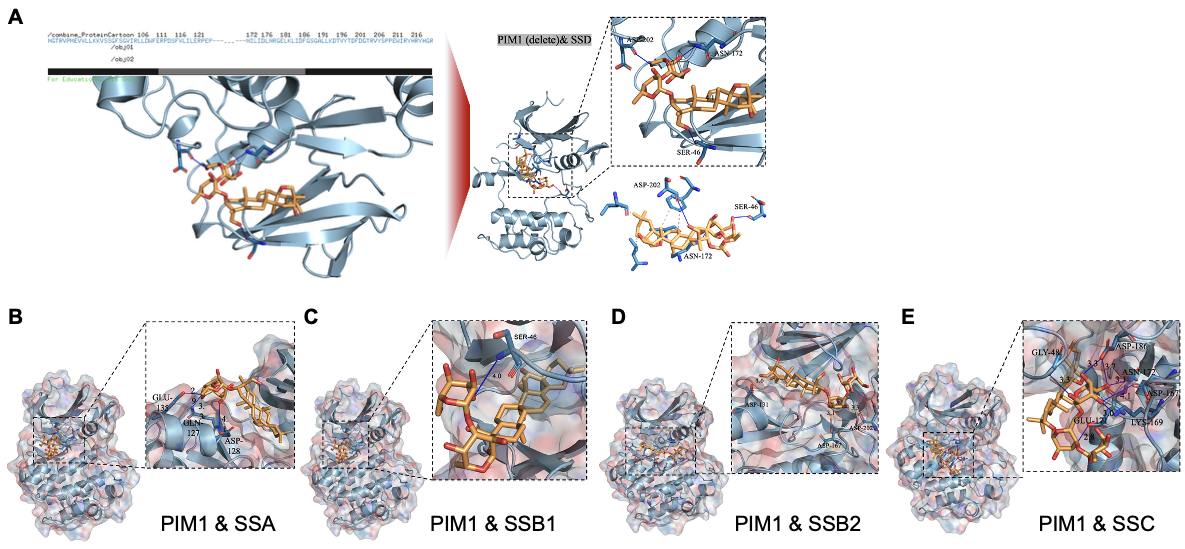


**Figure S7.** **Molecular docking analysis of SSDs with wild-type and mutant PIM1.** (A) Molecular docking analysis of SSDs with mutant PIM1. (B-E) Molecular docking analysis of SSA, SSB1, SSB2, and SSB with PIM1.

**Figure S8**


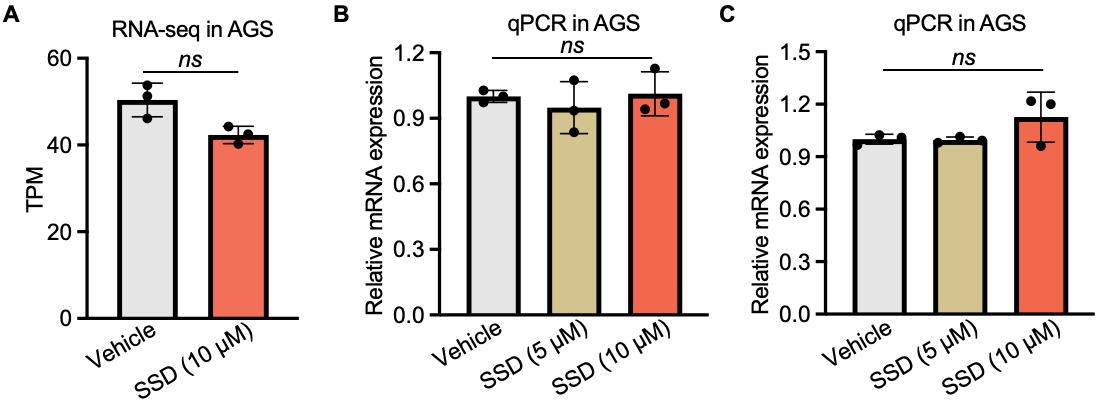


**Figure S8. Relative mRNA expression correlation of PIM1 after SSD treatment.** The relative mRNA expression of *PIM1* was determined by transcriptome analysis in AGS and qPCR in AGS and HGC-27 after cells were treated with vehicle or SSD.

**Figure S9**

**Figure S9. The binding affinity of SSD with PIM families and their expression profile in gastric cancer cells.** (A) Molecular docking of SSD with PIM1, PIM2, and PIM3. LB, lowest binding energy. (D and E) The relative mRNA expression PIM1, PIM2, and PIM3 in different gastric cancer cells was determined by transcriptome analysis (D) and qPCR (E).

**Figure S10**

**Figure S10. Inhibition of STAT3 has no significant impact on the anti-tumor efficacy of SSD.** (A) Molecular docking of SSD with STAT3. (B and C) CCK-8 assays were employed to determine the IC_50_ of STAT3 inhibitors, including Napabucasin and C188-9. (D and E) CCK-8 assays were carried out after cells were treated with STAT3 inhibitors Napabucasin (0.2 μM) and C188-9 (10 μM) or in combination with SSD (10 μM). FC, fold change in AGS (D) and HGC-27 cells (E).

**Table S1. Primers for RT-qPCR**

| **Primer name** | **5' to 3'** |
| --- | --- |
| qCYP1A1-F1 | GAAATCCCACCAGACCCCAG |
| qCYP1A1-R1 | CCCTGGCCTGGATTTCTCTG |
| qCYP1A1-F3 | CTGCTGGCTCATCCTTGACA |
| qCYP1A1-R3 | CTGGGTAATCAGGGCCTCAA |
| qCYP1A1-F2 | GCTCCAGCCCCAAAGGATAG |
| qCYP1A1-R2 | CAAGGGGCGTTGTGTCTTTG |
| qCYP1A1-F4 | AAGGGTTGGGTAGGTAGCGA |
| qCYP1A1-R4 | CCACCAAGAACTGCTTAGCC |
| qSNU13-F | CAGCCATGACTGAGGCTGAT |
| qSNU13-R | CCCCTGTTGAGGGTTTTGGT |
| qU2AF1-F | TGGGAAATGGCGGAGTATCTG |
| qU2AF1-R | TCAAGAGGGCAATGGTCTGG |
| qSNRPG-F | TTCCAAGGCATCTGTGAGCC |
| qSNRPG-R | TCCTTGGACATGTCTGCCAC |
| qPRPF38B-F | TTCTCGCGGTCTGGGTTTC |
| qPRPF38B-R | GGACCCTACTCCATCGAACG |
| qSNRPD2-F | ACCATCATGAGCCTCCTCAAC |
| qSNRPD2-R | ATGTTGCAGTGCCTATCGAA |
| qSNRPB-F | GGCGGGTATCAGAGCCATC |
| qSNRPB-R | TCAAAAGCCTTGAAGGTGCC |
| qSRSF7-F | GTCAGCTTTTGCGTCACTCG |
| qSRSF7-R | CACCTTGGTTTCTCCTCCGT |
| qACIN1-F | CCCGCTGTCCTCATTCAGTT |
| qACIN1-R | TGGCACTTTCTCTGCTGGAG |
| qSF3B6-F | CGAGCTCCGGAGGAATTTCA |
| qSF3B6-R | TCGAATGTTCGCCCTCTTGG |
| qSNRNP70-F | AAGCCGAAGCAGGAGTTGTT |
| qSNRNP70-R | GGAACTGGGTCATCTTGCCA |
| qSNRPD1-F | ATTCTGTGGCACCTACCCAC |
| qSNRPD1-R | ACCAGGTACCTAAGACTCCGT |
| qACTB-F | ACCGCGAGAAGATGACCCA |
| qACTB-R | GGATAGCACAGCCTGGATAGCAA |

**Table S2. gRNA oligos for CRISPR-Cas13**

| **Oligos name** | **5' to 3'** |
| --- | --- |
| CYP1A1-casRx-g1a-F | AAACGATTACCCAGAATACCAGACAG |
| CYP1A1-casRx-g1a-R | AAAACTGTCTGGTATTCTGGGTAATC |
| CYP1A1-casRx-g1b-F | AAACTTCTCACTTAACACCTTGTCGA |
| CYP1A1-casRx-g1b-R | AAAATCGACAAGGTGTTAAGTGAGAA |
| CYP1A1-casRx-g1c-F | AAACACAGAAGATGACAGAGGCCAGAA |
| CYP1A1-casRx-g1c-R | AAAATTCTGGCCTCTGTCATCTTCTGT |
| CYP1A1-casRx-g2a-F | AAACCCAGTAAGTTCAGAGATGCAGAG |
| CYP1A1-casRx-g2a-R | AAAACTCTGCATCTCTGAACTTACTGG |
| CYP1A1-casRx-g2b-F | AAACACAATCATTGCATTGATCCTCCT |
| CYP1A1-casRx-g2b-R | AAAAAGGAGGATCAATGCAATGATTGT |

| **Table S3. SSD predicted targets with different target prediction platforms** | | |
| --- | --- | --- |
| **SwissTargetPred** | **Super-PRED** | **SwissTargetPred** |
| STAT3 | APEX1 | VTDB |
| IL2 | GPR55 | ALBU |
| PTAFR | NFKB1 | BMP2 |
| PPM1B | GPR6 | AK1C2 |
| PPP1CC | ADORA1 | APOA2 |
| PPP2CA | BLM | STS |
| PPP2R5A | KDM1A | CASP7 |
| PFKFB3 | CLK4 | MK01 |
| TTL | KLF5 | CAH2 |
| HSD11B2 | NTRK3 | AOFB |
| HSD11B1 | SCN2A | ITAL |
| S1PR3 | NR3C2 | LPPL |
| S1PR1 | PIK3CA | CAH12 |
| RORC | PDE3A | GSTP1 |
| GABRA5 | TDP1 | FCAR |
| ADORA1 | FAAH | PYRD |
| ADORA2B | TOP2A | TTHY |
| ADORA3 | MTOR | PRGR |
| GPR119 | LTA4H | STK6 |
| JUN | CDK4 | GLCM |
| VEGFA | CTSD | HCK |
| FGF1 | BCHE | DHB11 |
| FGF2 | HSD17B10 | TGFR2 |
| IKBKE | FPR2 | MK10 |
| FASN | ACACA | CAH1 |
| TBK1 | DUSP3 | BACE1 |
| CXCR1 | C5AR1 | SEPR |
| PLA2G1B | HDAC2 | PDE4B |
| HDAC6 | ABL1 | ICAM2 |
| HDAC2 | GPRIN1 | CHLE |
| NCOR2 HDAC3 | IDO1 | EGFR |
| HDAC5 | ADAM10 | KIF11 |
| HDAC8 | S1PR5 | AMPM1 |
| HDAC1 | CDK1 | MMP13 |
| HDAC11 | PDGFRA | CFAB |
| MET | OPRK1 | MK08 |
| HDAC10 | CNR2 | LEG7 |
| GLRA1 | CCNE1 | FKB1A |
| GLRA2 | GRIA2 | CATD |
| ATP1A1 | MAOA | CHK1 |
| F10 | AKR1C3 | CATL2 |
| BCL2L1 | MC4R | MMP3 |
| DHODH | SCN3A | ANGI |
| KDR | ADAM17 | PNPH |
| RAP1A | CACNA1B | RORA |
| PDE4D | OPRM1 | EPHB4 |
| PTPN1 | AURKB | CLPP |
| ITGB1 ITGA4 | AR | QPCT |
| PTGS2 | PRMT1 | NR1H2 |
| F2RL1 | CCR1 | CASP3 |
| HTR4 | NFE2L2 | DDX6 |
| DRD4 | MAP3K14 | PH4H |
| PDE4B | PIK3R1 | AMPM2 |
| GRM2 | CHUK | THRB |
| PLK1 | TLR4 | DHI1 |
| PTPN11 | SCN4A | ADA17 |
| TYMS | FPR3 | EST1 |
| PIM1 | SCN9A | ESR1 |
| PIM2 | ACACB | WASP |
| PIM3 | ABCB1 | PTN11 |
| TLR7 | METAP2 | SHBG |
| AKT2 | HSP90AA1 | CATS |
| PTPN22 | PARG | PDE3B |
| AKT1 | CACNA1H | PPARG |
| GSK3B | ITK | ERG7 |
| HIF1A | HTR2A | SRC |
| OPRK1 | SCD | ISG20 |
| FLT1 | CDK2 | HS90A |
| MTNR1A | PDPK1 | BRAF1 |
| MTNR1B | PTK2B | TYSY |
| EGFR | TFPI | UROK |
| HRH1 | NR1I2 | ANPRC |
| CDK2 | CSNK2B | CCNA2 |
| CCR3 | TRIM24 | DYR |
| PRKCD | CFTR | MCR |
| PRKCB | CASP8 | ST14 |
| PRKCE | SLC6A5 | BMP7 |
| PRKCH | PRCP | ANXA5 |
| PRKCQ | HTR2C | CACP |
| DGAT1 | PDGFRB | ALDR |
| HSP90AA1 | SLC9A1 | FA10 |
| DCK | FFAR4 | NR1H4 |
| CTSK | PSMB9 | PDE4D |
| MMP13 | SLC1A2 | CALM |
| LTB4R | CXCR4 | BAG1 |
| BCL2 | TDO2 | CFAD |
| CASP1 | SLC1A3 | ADHX |
| MDM2 | ICMT | ZA2G |
| MAPK14 | STAT3 | ANDR |
| MMP2 | QRFPR | MMP7 |
| MMP8 | F11 | CATB |
| FANCF | TLR8 | CMA1 |
| PLA2G4A | VDR | ADH1B |
| CHEK1 | TMPRSS6 | FABP5 |
| LGALS4 | PDE7A | LCK |
| LGALS3 | HDAC1 | NOS3 |
| LGALS8 | HSP90AB1 | PTN1 |
| ABCC8 | ACVRL1 | GSK3B |
| ACHE | PTPN2 | TYPH |
| FAAH | RXFP1 | IMPA1 |
|  | BRD4 | FNTA |
|  | F7 | MMP8 |
|  | CNR1 | TGFR1 |
|  | FLT1 | FABP4 |
|  | F13A1 | DUS6 |
|  | S1PR2 | DCAM |
|  | PIK3CB | HXK1 |
|  | SOAT1 | PDE5A |
|  | PLA2G2A | CATK |
|  | SPHK1 | A1AT |
|  | TACR1 | MET |
|  | NOTUM | PPARA |
|  | DPP8 | DPP4 |
|  | SLC40A1 | RENI |
|  | KCNK9 | FABP6 |
|  | KDR | MIF |
|  | CYSLTR2 | RET4 |
|  | PIK3CG | CDD |
|  | CHRM5 | DHSO |
|  | GPR35 | ERR3 |
|  | HSD11B2 | ALDH2 |
|  | CAPN1 | KC1G2 |
|  | SIRT2 | MDM2 |
|  | NOX1 | PDK2 |
|  | CREBBP | PTGD2 |
|  | PROC | LEG2 |
|  | HDAC11 | KSYK |
|  | ATG4B | AK1C3 |
|  | RORB | G6PI |
|  | SLC1A1 | MMP12 |
|  | NPC1 | SAHH |
|  | HPRT1 | JAK3 |
|  | MMP7 | GALK1 |
|  | WDR5 | CTNA1 |
|  | PSMB1 | TGM3 |
|  | CDC25C | PLGF |
|  | CBX4 | NR1H3 |
|  | PLAT | NR1I3 |
|  | FPR1 | PAK6 |
|  | EGFR | NQO1 |
|  | CHRM1 | IGF1R |
|  | PDE3B | ACK1 |
|  | TACR2 | RXRA |
|  | KCNA5 | AKT1 |
|  | HRH4 | MMP2 |
|  | RORC | PAK7 |
|  | HDAC5 | IMDH2 |
|  | KDM4C | CP2C9 |
|  | HDAC10 | NCS1 |
|  | FABP4 | BST1 |
|  | SERPINE1 | HMDH |
|  | PRMT6 | FABPH |
|  |  | IL2 |
|  |  | CBR1 |
|  |  | PK3CG |
|  |  | PDPK1 |
|  |  | FABP7 |
|  |  | AMYP |
|  |  | FGFR1 |
|  |  | S10A9 |
|  |  | GCR |
|  |  | PPAP |
|  |  | CP2C8 |
|  |  | HYES |
|  |  | RAB5A |
|  |  | DCK |
|  |  | LEG3 |
|  |  | MAOM |
|  |  | ELNE |
|  |  | CDK6 |
|  |  | CLK1 |
|  |  | MP2K1 |
|  |  | ARGI2 |
|  |  | ZAP70 |
|  |  | CD1A |
|  |  | ALDOA |
|  |  | CCNT1 |
|  |  | RB11A |
|  |  | XIAP |
|  |  | FA7 |
|  |  | RARG |
|  |  | JAK2 |
|  |  | KTHY |
|  |  | KPCT |
|  |  | GLYC |
|  |  | B3GA1 |
|  |  | C1S |
|  |  | HINT1 |
|  |  | TPH1 |
|  |  | IMDH1 |
|  |  | CCL5 |
|  |  | NR1I2 |
|  |  | NGAL |
|  |  | MMP9 |
|  |  | TGM2 |
|  |  | NOS2 |
|  |  | ERBB4 |
|  |  | GRB2 |
|  |  | PADI4 |
|  |  | TPIS |
|  |  | PNMT |
|  |  | CSK |
|  |  | DPEP1 |
|  |  | CD5R1 |
|  |  | LGUL |
|  |  | LYAM3 |
|  |  | TIE2 |
|  |  | ST1E1 |
|  |  | KAT1 |
|  |  | PIM1 |
|  |  | MTAP |
|  |  | MAPKAPK2 |
|  |  | PPIA |
|  |  | TREM1 |
|  |  | SULT2A1 |
|  |  | KDR |
|  |  | CDK2 |
|  |  | TNNC1 |
|  |  | HSD17B1 |
|  |  | HDAC8 |
|  |  | NUDT9 |
|  |  | MAPK14 |
|  |  | SEC14L2 |
|  |  | AKR1C1 |
|  |  | THRB |
|  |  | TTPA |
|  |  | PPP5C |
|  |  | ADH1C |
|  |  | CHIT1 |
|  |  | ADK |
|  |  | PYGL |
|  |  | IGLV2-8 |
|  |  | LOC100652777 |
|  |  | NQO2 |
|  |  | APCS |
|  |  | HSPA8 |
|  |  | PLK1 |
|  |  | GSR |
|  |  | CYP19A1 |
|  |  | MTHFD1 |
|  |  | PRKACA |
|  |  | Esr2 |
|  |  | PLA2G2A |
|  |  | C1R |
|  |  | SOD2 |
|  |  | CSNK2A1 |
|  |  | PARP1 |
|  |  | TRAPPC3 |
|  |  | ITK |
|  |  | PPARD |
|  |  | ABO |
|  |  | PNPO |
|  |  | REG1A |
|  |  | SULT2B1 |
|  |  | LDHB |
|  |  | HNF4G |
|  |  | UCK2 |
|  |  | THRA |
|  |  | GSTA1 |
|  |  | GM2A |
|  |  | RNASE4 |
|  |  | OTC |
|  |  | ESRRA |
|  |  | HNMT |
|  |  | ATOX1 |
|  |  | MAN1B1 |
|  |  | CPB1 |
|  |  | FGFR2 |
|  |  | FHIT |
|  |  | BLVRB |
|  |  | RAC1 |
|  |  | C8G |
|  |  | RHEB |
|  |  | CRABP2 |
|  |  | HEXB |
|  |  | YARS1 |
|  |  | FECH |
|  |  | RNASE3 |
|  |  | PCK1 |
|  |  | BCAT2 |
|  |  | PRKACA |
|  |  | RHOA |
|  |  | SDS |
|  |  | SRM |
|  |  | SSE1 |
|  |  | LYZ |
|  |  | UMPS |
|  |  | APRT |
|  |  | GNPDA1 |
|  |  | BIRC7 |
|  |  | NMNAT1 |
|  |  | ADAM17 |
|  |  | HAGH |
|  |  | PSAP |
