# Supplementary material for: Saikosaponin‑D triggers cancer cell death by targeting the PIM1/c-Myc axis to reprogram oncogenic alternative splicing
Source: Cell Death Discov. 2025 Oct 6;11:427. doi: 10.1038/s41420-025-02729-w (PMC12501015; doi:10.1038/s41420-025-02729-w)

Corresponding to Figure 5G

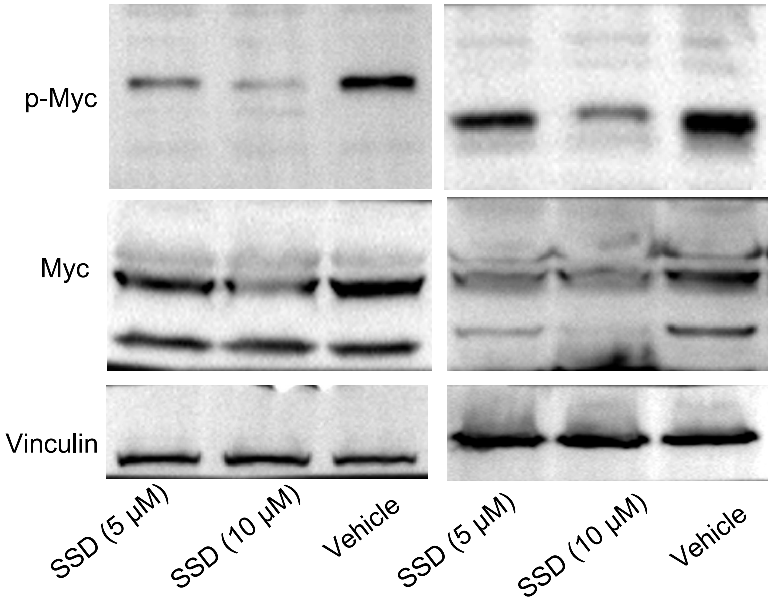

Corresponding to Figure 5H

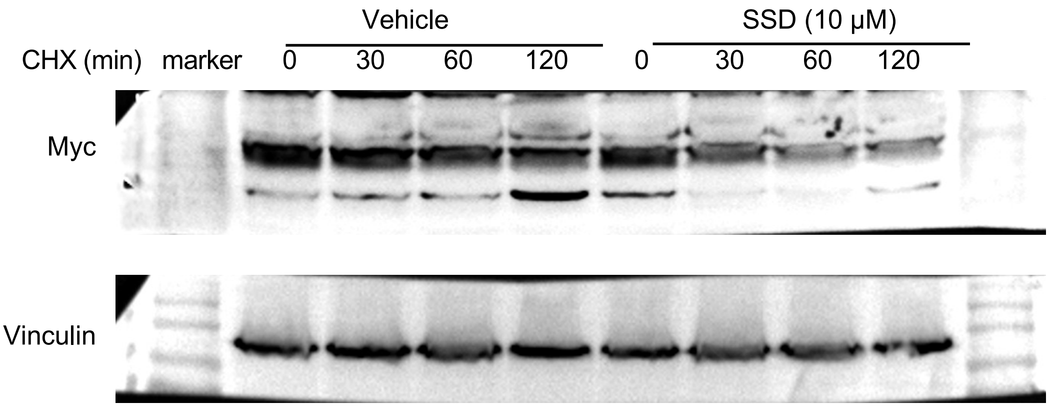

Corresponding to Figure 6E

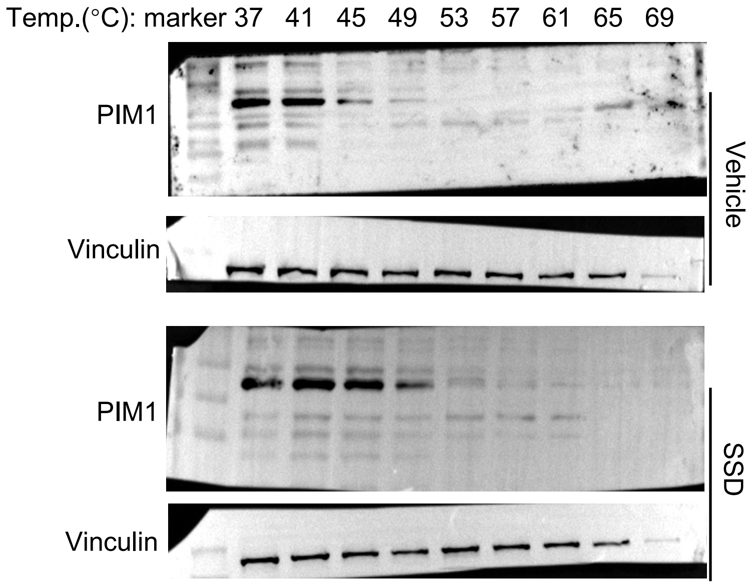

Corresponding to Figure 6G

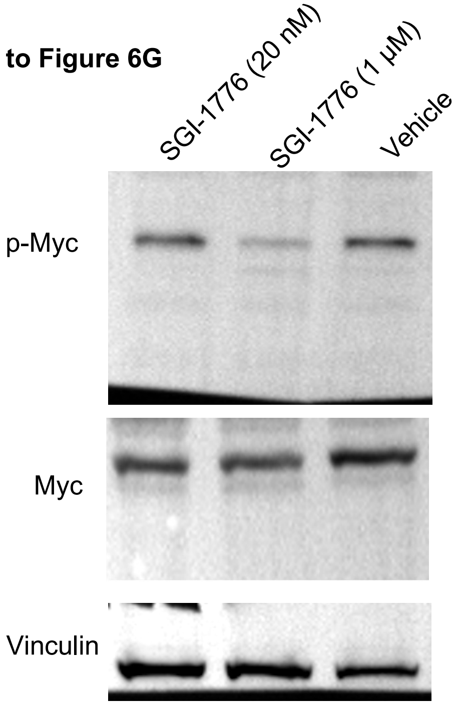

Corresponding to Figure 6I

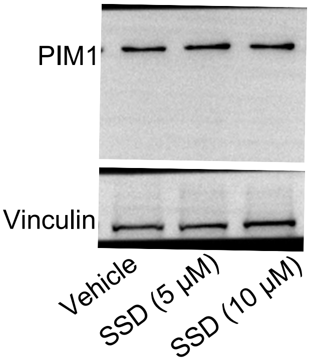

Corresponding to Figure 6J

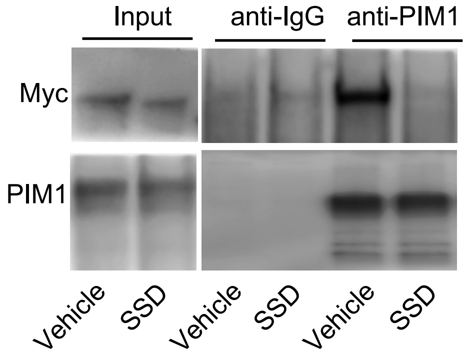

Supplement: Supplementary file 2 — Raw Image for WB [file 41420_2025_2729_MOESM2_ESM.pdf]
